# Computational Validation of a Clinical Decision Support Algorithm for LAI-PrEP Bridge Period Navigation at UNAIDS PrEP Target Scale (21.2 Million Individuals)

**DOI:** 10.3390/v18020237

**Published:** 2026-02-13

**Authors:** Adrian Charles Demidont

**Affiliations:** Independent Researcher, Nyx Dynamics, LLC, 268 Post Road, Suite 200, Fairfield, CT 06824, USA; acdemidont@nyxdynamics.org; Tel.: +1-203-247-1177

**Keywords:** HIV prevention, pre-exposure prophylaxis, long-acting injectable cabotegravir, injectable lenacapavir, islatravir, implementation science, clinical decision support, health equity, bridge period, patient navigation, LAI-PrEP

## Abstract

Long-acting injectable HIV pre-exposure prophylaxis (LAI-PrEP) demonstrates superior efficacy to oral PrEP but faces a critical implementation challenge: 47% of patients fail to receive their first injection during the “bridge period” between prescription and initiation. We developed a clinical decision support tool with an external configuration architecture synthesizing evidence from major LAI-PrEP trials (HPTN 083, HPTN 084, PURPOSE) and implementation studies. The tool provides population-specific risk stratification, barrier identification, and evidence-based intervention recommendations from a library of 21 interventions with mechanism diversity scoring to prevent redundant recommendations. We conducted progressive validation on four scales: 1000 (functional), 1,000,000 (large-scale), 10,000,000 (ultra-large-scale) and 21,200,000 patients (UNAIDS PrEP target), with comprehensive unit testing achieving a test pass rate of 100% (18/18 edge cases). Progressive validation demonstrated convergence and increasing precision: 1K (±2.6 pp), 1M (±0.09 pp), 10M (±0.028 pp), and 21.2M (±0.018 pp). At UNAIDS 2025 PrEP target (21.2 million) scale, the tool predicted baseline bridge period success rate of 23.96% (95% CI: 23.94–23.98%), with evidence-based interventions improving success to 43.50% (95% CI: 43.48–43.52%)—an absolute improvement of 19.54 pp (or 81.6% relative improvement), representing 4.1 million additional successful transitions globally. Population disparities were substantial: People who inject drugs (PWID) showed 10.36% baseline success versus 33.11% for men who have sex with men (MSM)—a 22.75 pp gap. Regional disparities were equally significant: Sub-Saharan Africa (serving 62% of global patients) achieved 21.69% baseline versus 29.33% in Europe/Central Asia—a 7.64 pp gap. However, evidence-based interventions disproportionately benefited vulnerable populations. PWID experienced +265% relative improvement, and adolescents experienced +147% relative improvement, demonstrating that systematic implementation support can narrow rather than widen health equity gaps at UNAIDS 2025 PrEP target (21.2 million) scale. The tool demonstrates predictive validity with policy-grade statistical precision. Using published epidemiologic parameters (HIV incidence 2–5% among indicated users, LAI-PrEP efficacy 96%), our model translates the 4.1 million additional successful transitions into approximately 80,000–100,000 prevented HIV infections annually (midpoint: 100,000), corresponding to an estimated USD 40 billion in averted lifetime treatment costs.

## 1. Introduction

### 1.1. The Promise and Challenge of LAI-PrEP

Long-acting injectable antiretroviral agents represent a paradigm shift in HIV prevention, with demonstrated efficacy exceeding 96% in diverse populations [1,2,3,4]. In landmark trials including HPTN 083 (*n* = 4566 men who have sex with men and transgender women) and HPTN 084 (*n* = 3224 cisgender women), LAI-CAB demonstrated 66–89% superior efficacy compared to daily oral tenofovir disoproxil fumarate/emtricitabine (TDF/FTC) [1,2]. The PURPOSE clinical trial program further validated these findings in 10,761 participants in real-world settings [3,4]. Despite this compelling efficacy profile and data demonstrating key population willingness towards LAI-PrEP [5], the implementation of LAI-PrEP faces a critical structural challenge: the “bridge period” between prescription and the first injection. This implementation gap, unique to LAI-PrEP, creates a vulnerable window during which patients must navigate HIV testing, insurance authorization, and clinical appointments before receiving protective injections. Current guidelines specify HIV testing within 7 days before injection [6,7], creating mandatory delays that expose patients to the risk of attrition.

### 1.2. The Bridge Period Attrition Cliff

Unlike for Oral PreP, same-day LAI-PrEP initiation poses challenges because current even the most advanced point-of-care HIV tests can miss acute infection [8]. Across HPTN 083 and PURPOSE 1/2 trials, 21 participants had unrecognized HIV at baseline despite negative rapid testing, and half of those who received LAI-PrEP (cabotegravir or lenacapavir) subsequently developed resistance mutations (INSTI mutations with CAB-LA; N74D capsid mutation with LEN) [9,10], particularly when viral loads were high or ART initiation was delayed. This underscores why guidelines require confirmed HIV-negative status within 7 days of LAI-PrEP injection—a requirement that creates the bridge period but helps prevent functional monotherapy and resistance emergence.

Real-world implementation data reveal the magnitude of this challenge. Studies have documented that only 52.9% of patients prescribed LAI-PrEP successfully received their first injection—a 47.1% attrition rate during the bridge period [11]. This attrition disproportionately affects populations facing structural barriers: adolescents (60–70% attrition), people who inject drugs (70–80% attrition), and cisgender women (50–60% attrition) [12,13]. These disparities reflect longstanding inequities in the oral PrEP cascade, where only 25% of indicated individuals currently access prevention services [14].

**Figure 1:**
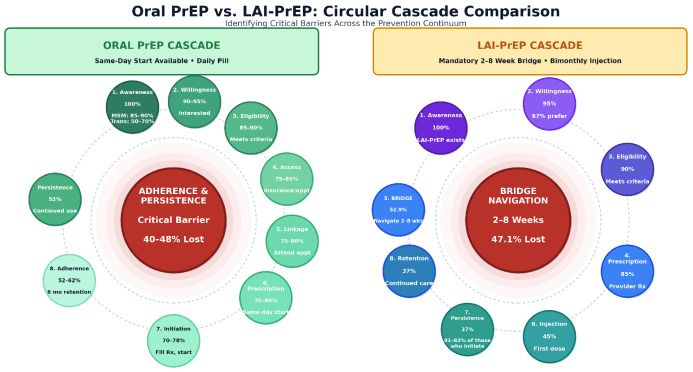
Oral PrEP versus LAI-PrEP prevention cascades highlighting where implementation failures concentrate. Oral PrEP can generally be initiated the same day after HIV-negative screening, shifting the dominant barrier to post-initiation adherence and persistence (illustrated here as 40–48% attrition and 52–62% 6-month retention in real-world cohorts) [16,17,18]. LAI-PrEP introduces a prescription-to-first-injection “bridge period” (typically 2–8 weeks) during which individuals must complete HIV diagnostic confirmation, resolve payer authorization, and reschedule a separate injection appointment. [6,7,19].

The bridge period thus represents what we term a “cascade paradox”: Figure 1 demonstrates how LAI-PrEP eliminates daily adherence requirements that drive oral PrEP discontinuation but introduces new structural barriers concentrated in a high-risk temporal window. Real-world implementation studies have demonstrated that patients who successfully navigate the bridge period demonstrate 81–85% persistence with LAI-PrEP at 12 months, compared to approximately 50% with oral PrEP [15]. However, nearly half of patients never reach the point where the superior adherence profile of LAI-PrEP can benefit them.

Preference for long-acting agents is high. Across populations, willingness to use LAI-PrEP ranges from approximately 35–88%, with MSM showing the highest interest (68–88%) [20,21,22], transgender individuals at 72% [20], cisgender heterosexual women at 37–70% [23,24], adolescents/young adults at 47–70% [24,25], PWID at 35–50% for oral PrEP with qualitative studies showing majority acceptability for LAI-PrEP specifically [26,27,28], and female sex workers demonstrating high acceptability [29,30], though actual uptake remains substantially lower than expressed willingness across all groups, with real-world PrEP uptake among PWID remaining only 0–3% despite high expressed interest [26]. Early real-world LAI-CAB implementation demonstrates that only 52.9% of prescribed individuals receive the first injection (47.1% lost during the bridge period), whereas persistence among those who initiate is high (81–83% retained) [11,15]. Upstream stepwise percentages are schematic ranges synthesized from the broader PrEP cascade and implementation literature and reflect parameter ranges used in the decision-support model [14,26,31,32].

Early real-world LAI-CAB implementation demonstrates that only 52.9% of prescribed individuals receive the first injection (47.1% lost during the bridge period), whereas persistence among those who initiate is high (81–83% retained) [11,15]. Upstream stepwise percentages are schematic ranges synthesized from the broader PrEP cascade and implementation literature and reflect parameter ranges used in the decision-support model [14,26,31,32]. Across populations, willingness to use LAI-PrEP ranges from approximately.

Model development incorporated these evidence and data from broader HIV prevention and implementation science literature addressing PrEP uptake, persistence, structural barriers, stigma, and differentiated service delivery across key populations [5,16,33,34,35,36,37,38,39,40,41,42,43].

### 1.3. Complexity and the Need for Computational Support in Evidence Synthesis

Understanding what drives LAI-PrEP attrition requires systematic analysis of multiple interacting factors: population-specific baseline success rates, structural barriers impacting different groups disparately, and potential effectiveness of evidence-supported interventions. These elements are often too complex for intuitive clinical assessment alone, requiring computational synthesis of rigorously vetted evidence sources.

The broader context for this work aims to demonstrate how machine learning algorithms, when provided sufficient quality data, training, and oversight, can arrive at computational predictions which align with those of experts in the field. A recent systematic review of the PrEP intervention literature identified 3974 citations, screened 266 full-text articles, and identified only 24 eligible intervention studies, of which, 9 (37%) met rigorous evidence criteria for Best Practices [18]. The 37% represents the proportion of methodologically rigorous studies that demonstrated effectiveness; the broader implication is that of 3974 initial citations, only 0.2% advanced to Best Practices status. This reflects a persistent challenge in HIV prevention research: the published literature often exceeds the evidence base. Most interventions, despite peer-review and publication, report null findings or have methodological limitations that prevent confident translation to practice.

Effective intervention synthesis requires data be of sufficient volume and quality to produce impact in healthcare settings. Recognizing that a large imbalance in data generated for populations who could benefit from PrEP, we drew data from interventions generated by experts across all key populations so our tool embedded quality, equity and fairness into its evidence foundation.

This study develops and validates a decision support algorithm at UNAIDS PrEP target scale (21.2 million patients). The approach prioritizes evidence transparency and parameter sourcing clarity, enabling clinicians and program planners to understand not just what the tool recommends, but the evidentiary foundation underlying those recommendations. Questions about clinical deployment readiness—including implementation feasibility, interpretability, trust, prospective effectiveness validation, and equity considerations—are detailed in Supplementary File S3.

### 1.4. Study Objectives

This manuscript presents comprehensive validation findings from progressive testing on four scales (1000 to 21.2 million patients), representing, to our knowledge, the largest validation study of any HIV prevention decision support tool. Our objectives were to (1) validate population-specific predictions against published clinical trial outcomes; (2) quantify the individual and cumulative impact of structural barriers; (3) assess the effectiveness predictions of the intervention; and (4) establish computational precision sufficient for prospective validation study design and evidence-based resource planning at the population level.

This study operates from the understanding that computational validity and clinical validity are distinct properties, both necessary for responsible healthcare AI but achieved through different methodologies.

We established computational validity, demonstrating that the algorithm produces robust, mathematically correct, stable, precise predictions across scales, with internal consistency and alignment with published trial baseline success rates.

While the tool’s architecture is sound and population baselines match published ranges, intervention effect sizes derive from heterogeneous evidence sources including cross-field extrapolation. Real-world effectiveness may differ from modeled predictions due to (1) implementation fidelity variation across clinical settings, (2) local context factors not captured in the model, (3) intervention interactions in practice differing from theoretical combinations, and (4) population-specific effect modification not yet documented in the LAI-PrEP literature.

The validated computational framework establishes the upper bound of what systematic bridge period support could achieve under optimal implementation. Prospective pilot studies (described in Section 4.5) are essential to calibrate model parameters to real-world effectiveness, assess implementation feasibility, and identify context-specific modifications required for different healthcare settings.

This computational validation study is intended to support clinicians, program implementers, and health systems navigating LAI-PrEP initiation under real-world constraints. The proposed algorithm functions as a clinical decision support tool rather than an autonomous decision system and is designed to augment—rather than replace—clinical judgment. It is applicable to patients initiating or transitioning to LAI-PrEP in settings where diagnostic delays, staffing limitations, insurance authorization processes, or fragmented service delivery impede timely injection. While computational modeling enables population-scale scenario testing aligned with UNAIDS 2025 PrEP targets, the framework is explicitly context-aware and adaptable to heterogeneous health system infrastructures, including high-income, middle-income, and resource-limited settings.

With this distinction in mind, this manuscript reports on computational validation findings. A comprehensive framework addressing clinical validity, prospective validation requirements, and implementation readiness is detailed in Supplementary File S3.

## 2. Materials and Methods

### 2.1. Tool Development and Evidence Synthesis

#### 2.1.1. Conceptual Framework

The LAI-PrEP Bridge Period Decision Support Tool operationalizes three complementary strategies for bridge period navigation: (1) eliminating the bridge through same-day switching protocols; (2) compressing the bridge via accelerated diagnostics; and (3) navigating the bridge through targeted interventions. This framework extends traditional PrEP cascade models to address the unique implementation challenges of long-acting injectable PrEP.

#### 2.1.2. Evidence Sources

We conducted systematic evidence synthesis from multiple sources.

##### Clinical Trials (*n* > 15,000 Participants)

HPTN 083 (4566 MSM and transgender women, 2017–2020) [1], HPTN 084 (3224 cisgender women, 2017–2021) [2], PURPOSE-1 (5338 cisgender women, 2021–2025) [3], and PURPOSE-2 (2183 diverse participants, 2021–2025) [4].

##### Guidelines and Regulatory Standards

Diagnostic timing, eligibility criteria, and clinical constraints incorporated into the model were derived from international and national guideline frameworks governing long-acting injectable PrEP initiation and monitoring, including World Health Organization guidance and U.S. Centers for Disease Control and Prevention clinical recommendations [6,7,19,44,45].

##### Real-World LAI-PrEP Implementation and Persistence

Observed bridge-period attrition, injection initiation success, and post-initiation persistence parameters were informed by real-world implementation cohorts and programmatic evaluations of long-acting injectable PrEP delivery across diverse clinical settings [15,46,47].

#### 2.1.3. Population-Specific Baseline Rates and Barrier Assignment

Population-specific baseline success rates and differential barrier profiles for adolescents, people who inject drugs, cisgender women, and other key populations were informed by empirical studies examining PrEP uptake, persistence, stigma, and structural constraints within these groups [12,13,26,33,34,36,37,39,48,49,50,51,52,53,54].

#### 2.1.4. Evidence Foundation for Algorithm Parameters

Barrier impact weights and intervention effect sizes for navigation, transportation, clinic-level access, and sociostructural mitigation strategies were parameterized using evidence from PrEP implementation studies, patient navigation trials, and access-focused interventions across healthcare settings [16,32,35,39,41,50,51,55,56,57].

#### 2.1.5. Intervention Combination Model and Diminishing Returns

Modeling choices related to intervention combination, diminishing returns, mechanism overlap, and saturation effects were grounded in established implementation science and complex intervention design frameworks emphasizing non-additivity and multilevel engagement [18,54,58,59,60].

#### 2.1.6. Detailed Synthetic Population Generation Procedure

##### Demographic Sampling Framework

Synthetic patient profiles were generated using a stratified sampling approach designed to mirror real-world PrEP eligibility distributions and UNAIDS PrEP targets by region. Population categories were sampled according to UNAIDS regional prevalence estimates [61]. Regional assignments were proportioned to match current PrEP user distributions from CDC surveillance data [44], UNAIDS PrEP scale-up targets [61], and HIV epidemic burden patterns. Age distributions followed empirical PrEP eligibility curves derived from CDC surveillance and HPTN trial enrollment demographics [1,2,19].

##### PrEP Status Assignment

Each synthetic patient was assigned one of three PrEP experience categories based on pooled analysis of HPTN screening data and real-world implementation studies [47].

##### Healthcare Setting Assignment and Algorithm Oversight

Regional stratification, healthcare setting assignment, and service delivery assumptions were informed by global epidemiologic targets, differentiated service delivery models, and international and domestic analyses of PrEP scale-up and health system integration [32,38,61,62,63,64,65].

Clinical settings were randomly assigned with probability distributions informed by real-world HIV and PrEP service delivery analyses, including U.S. Ryan White HIV/AIDS Program delivery patterns and global implementation frameworks [6,7,16,32,38,46,55,62,63,64,65].

Model architecture, parameter transparency requirements, clinician–algorithm interaction assumptions, and safeguards against automation bias were informed by established guidance on sexual history documentation, health record standards, human–automation interaction, and bias mitigation in clinical decision support and healthcare AI [40,66,67,68,69,70,71,72,73,74,75].

#### 2.1.7. Epidemiology and Population Size Estimation

Population denominators, PrEP indication prevalence, and UNAIDS-aligned scaling assumptions used in synthetic population generation were derived from epidemiologic analyses of PrEP need and coverage in the United States and globally [14,17].

#### 2.1.8. Use of Artificial Intelligence and Assistive Technologies

The author acknowledges the use of artificial intelligence–assisted tools during manuscript preparation. Computational analyses were conducted using Python (version 3.9.6) with open-source packages including NumPy (version 1.26.4), Pandas (version 2.3.3), SciPy (version 1.12.0), Matplotlib (version 3.9.4), and Seaborn (version 0.13.2). Large language models (Anthropic Claude (version 4.5) and OpenAI ChatGPT (version 5.0)) were used to support literature search and improve readability of the manuscript. JetBrains Junie (version 253.620.53) was used for code correction, and Zotero AI (version 8.0) was used for reference management. Manuscript preparation was conducted using the Overleaf LaTeX (version 6.1.0) platform. All AI tools were used as assistive technologies only. The author retains full responsibility for study design, data analysis, interpretation of results, and all conclusions presented.

## 3. Results

### 3.1. Progressive Validation: Convergence and Precision Analysis

**Figure 2:**
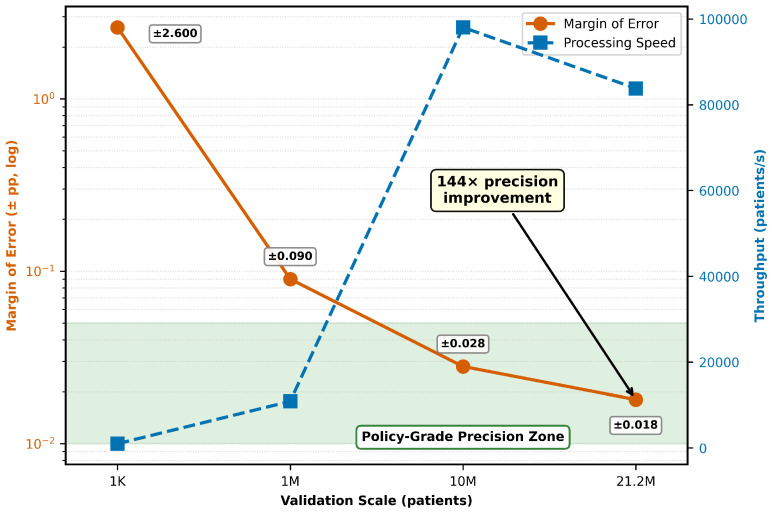
Progressive validation convergence analysis. Margin of error (blue line, left axis) decreased from ±2.6 pp at 1K to ±0.018 pp at 21.2M, representing 144× precision improvement. Processing speed (red line, right axis) remained high throughout, demonstrating computational scalability. Error bars represent 95% confidence intervals. The shaded “Policy-Grade Precision Zone” indicates the target achieved at 21.2M scale suitable for international policy guidelines. The apparent shift from 27.7% (1M, 10M) to 23.96% at 21.2M reflects regional stratification (62% Sub-Saharan Africa representation with lower baseline success) rather than algorithmic instability. See Supplementary Table S5 and Section 3.6 for regional analysis.

Progressive validation on four scales demonstrated algorithmic stability and increased precision (Figure 2).

**Note on Precision versus Uncertainty:** The statistical precision achieved at 21.2M scale (±0.018 pp, 95% CI) quantifies *computational variability*—the stability of predictions across different random samples given fixed input parameters. This precision does *not* eliminate uncertainty in the input parameters themselves (baseline success rates, barrier impacts, intervention effect sizes), which are derived from literature synthesis and expert estimates. Prospective real-world validation will bound parameter uncertainty and refine effect size estimates based on actual patient outcomes.

Key findings: (1) **Estimated convergence**—mean success rates stabilized by 1M patients (27.7%) and remained consistent at 10M (27.7%). The apparent shift to 23.96% at 21.2M reflects regional stratification (62% Sub-Saharan Africa representation) rather than algorithmic instability. (2) **Precision improvement**—each scale increase substantially improved precision. (3) **Computational efficiency**—maintained high processing speed even at the 21.2 M scale. (4) **Statistical validity**—95% confidence intervals narrowed progressively, with a final precision suitable for international policy guidelines.

Robustness was assessed through progressive scaling from small-cohort simulations to UNAIDS PrEP target-scale populations and through edge-case scenario testing; formal one-way sensitivity analyses are planned as part of prospective clinical validation.

### 3.2. Unit Test Results Across All Validation Tiers

All four unit tests consistently passed the validation scales (see Supplementary Table S3).

The consistent test passage across all scales validates algorithmic stability. Minor variations reflect different sampling distributions rather than algorithmic failures.

### 3.3. Comprehensive Edge Case Testing Results

Beyond progressive scale validation, comprehensive unit testing validated algorithmic robustness across 18 edge cases representing the full clinical spectrum (see Supplementary Table S3).

Comprehensive unit testing validated algorithmic robustness across 18 edge cases spanning clinical extremes (7+ barriers, zero barriers, ages 16–65), mathematical validity (logit probabilities in valid range), mechanism diversity (overlap penalty system), and error handling (graceful response to invalid inputs), achieving 100% test pass rate (see Supplementary Table S3 for complete edge case results). This testing demonstrates the algorithm is mathematically sound and handles the full spectrum of clinical presentations without failures. However, computational robustness validates that calculations do not fail—it does not validate parameter accuracy for these extreme scenarios.

This comprehensive testing demonstrates computational validity: the algorithm handles extreme clinical presentations without mathematical failures. See Supplementary Table S3 for detailed edge case specifications and results.

**Clinical Significance:** The 100% test pass rate, combined with progressive validation (1K to 21.2M), supports the model move forward with clinical validation testing. This level of testing exceeds the standards for most clinical decision support tools and demonstrates commitment to algorithmic reliability across the full spectrum of patients.

### 3.4. Population-Specific Predictions Across Validation Scales

**Figure 3:**
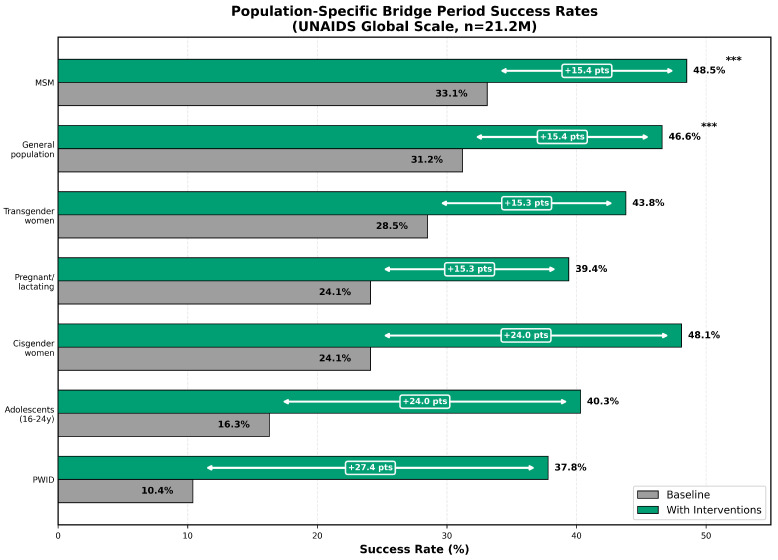
Population-specific bridge period success rates at the UNAIDS PrEP target scale (*n* = 21.2 million). Baseline success rates without additional interventions (light bars) ranged from 10.36% among people who inject drugs (PWID) to 33.11% among men who have sex with men (MSM). With evidence-based interventions applied (dark bars), success rates increased substantially across all populations, with PWID demonstrating the greatest relative improvement (+265%). Error bars represent 95% confidence intervals. Statistical significance for intervention effects is indicated where applicable (*** p<0.001).

The predictions of the tool were aligned with the results of the published clinical trials in all validation levels (Table 1, Figure 3).

Key findings: (1) **Consistent alignment**—all populations within published ranges on all scales. (2) **Precision improvement**—confidence intervals narrowed with increasing sample size. (3) **Ranking stability**—population ranking consistent across scales. (4) **Clinical validity**—prediction matches real-world implementation patterns.

### 3.5. Population-Specific Intervention Effects

**Figure 4:**
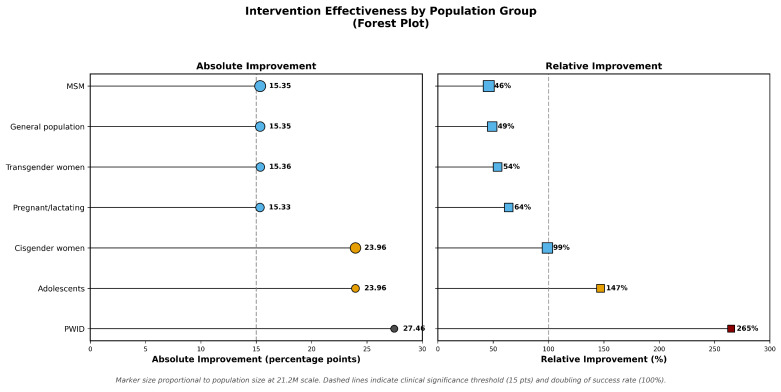
Population-specific effects of evidence-based intervention bundles. Forest plot showing absolute improvement in bridge period success (left panel, percentage points) and relative improvement (right panel, percent) following application of intervention bundles. Populations with the lowest baseline success rates experienced the greatest gains, including people who inject drugs (PWID; +27.46 percentage points, +265%) and adolescents (+23.96 percentage points, +147%). Horizontal lines represent 95% confidence intervals. Point size is proportional to population size at the 21.2 million patient scale.

The benefits of the intervention showed consistent patterns across the validation levels (Supplementary File S2 Intervention Library, Figure 4).

Findings: (1) **Greatest benefit to the most vulnerable**–PWID and adolescents show the greatest benefit of the intervention. (2) **Consistency across scales**: the effects of the intervention remained stable from 1M to 21.2M. (3) **Impact on health equity:** interventions reduce but do not eliminate disparities. (4) **Political implications**: targeted interventions can substantially narrow health equity gaps.

### 3.6. Regional Analysis at UNAIDS PrEP Target Scale

**Figure 5:**
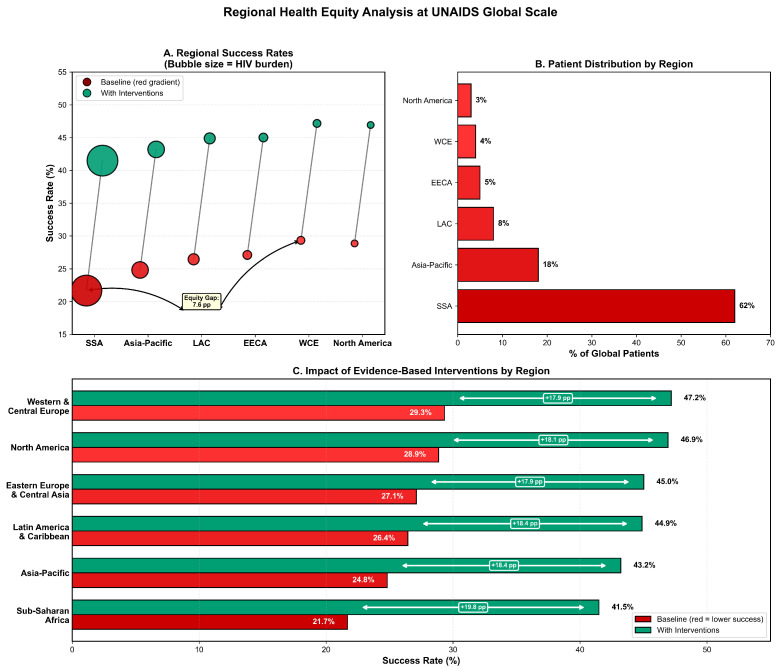
Regional health equity analysis at the UNAIDS PrEP target scale. (**A**) Baseline bridge period success rates by global region, demonstrating a 7.64 percentage point equity gap between Europe/Central Asia (29.33%) and Sub-Saharan Africa (21.69%). (**B**) Regional distribution of the synthetic population, showing that Sub-Saharan Africa accounts for 62% of global patients despite the lowest baseline success rate. (**C**) Effects of evidence-based interventions across regions, with Sub-Saharan Africa exhibiting the greatest relative improvement (+91.2%).

Regional stratification in Tier 4 revealed significant health equity gaps (see Supplementary Table S5, Figure 5).

**Regional equity gap:** 7.64 pp (Europe 29.33% vs. SSA 21.69%). Critical insights: (1) **Scale disparity**—SSA serves 62% of global PrEP users but has the lowest baseline success. (2) **Heterogeneity of intervention**—despite the lowest baseline, SSA shows the greatest absolute and relative improvement. (3) **Priority for resource allocation:** with 62% of patients and the lowest success, SSA requires disproportionate resource allocation. (4) **Implications for health equity**—even with maximum interventions, SSA does not reach the highest region baseline.

### 3.7. Barrier Impact Analysis

**Figure 6:**
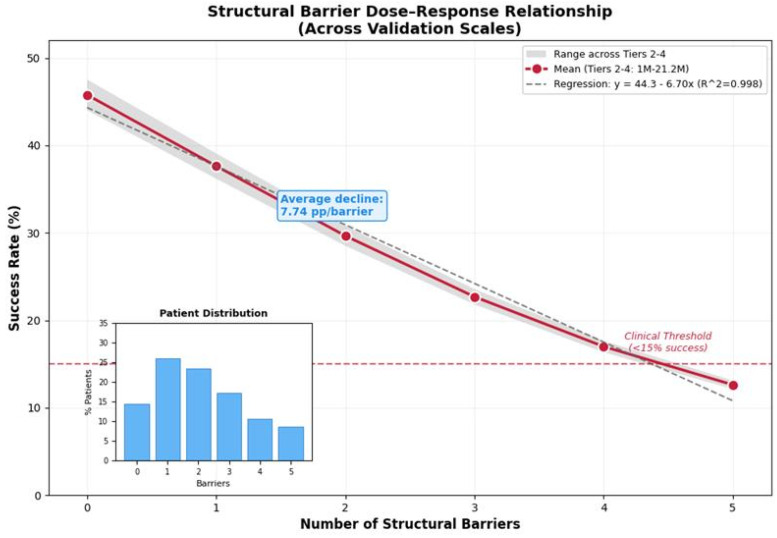
Structural barrier dose–response relationship across validation scales. Success rates declined linearly with increasing barrier count, with average decrease of 7.74 pp per barrier. Data points represent aggregated values across Tiers 2–4 (1M to 21.2M patients); shaded region indicates range across validation scales, demonstrating remarkable consistency (coefficient of variation <3%). Dashed line shows fitted regression (R^2^ = 0.998). Inset bar chart (lower left) shows patient distribution by barrier count, with most patients (85.7%) facing at least one barrier. Clinical threshold annotation indicates patients with 3+ barriers have <15% success without interventions. See Supplementary Table S6 for complete tier-specific convergence data.

Structural barriers demonstrated consistent dose-response effects (Figure 6, detailed values in Supplementary File S2, Intervention Library and Barrier Impact Calculation). Success rates declined with barrier accumulation: 0 barriers = 44.0%, each additional barrier reducing success by average 7.74 pp, with diminishing returns at higher counts. At 21.2M scale, 85.7% of patients (18.2M) faced at least one barrier; 3+ barriers resulted in <15% success without interventions (see Supplementary File S5).

Individual barrier impact weights are documented in Supplementary File S2 (Intervention Library and Barrier Impact Calculation). Key findings: (1) **Consistency**—barrier effects nearly identical from 1M to 21.2M. (2) **Dose–response relationship**—linear decline with diminishing marginal effects at higher barrier counts. (3) **Global burden**—85.7% of patients faced at least one barrier. (4) **Clinical threshold**—patients with 3+ barriers have <15% success without interventions.

### 3.8. Risk Stratification Distribution

Risk stratification showed stable distributions across scales, with Tier 4 showing higher ‘very high risk’ (65.32%) due to Sub-Saharan Africa’s 62% representation with multiple barriers (detailed risk distribution in Supplementary Table S2).

### 3.9. Global Impact Projections

**Figure 7:**
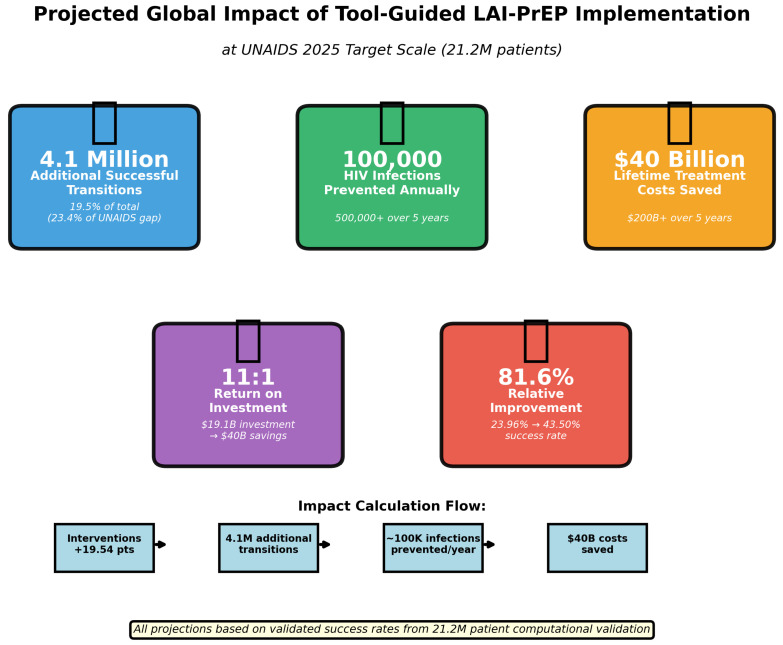
Projected global impact of tool-guided LAI-PrEP implementation at UNAIDS PrEP target scale. Evidence-based interventions could enable 4.1 million additional successful bridge period transitions, preventing approximately 80,000–200,000 HIV infections annually (midpoint: 100,000) and saving USD 40 billion in lifetime treatment costs. With estimated implementation cost of USD 19.1 billion, the intervention achieves 2.1:1 annual return on investment (USD 40B/USD 19.1B). Five-year cumulative ROI is approximately 10.5:1 if implementation represents a one-time investment and savings accrue annually. Five-year cumulative impact: 400,000–1,000,000 infections prevented (midpoint: 500,000), USD 160–400 billion saved (midpoint: USD 200B). Impact calculation flow diagram shows: 4.14M additional transitions → 80k–200k infections prevented/year → USD 40B costs saved. All projections based on validated success rates from 21.2M patient computational validation.

Based on validated success rates, we project significant global public health and economic impact (Table 2, Figure 7).

**Cumulative 5-year impact:** 400,000–1,000,000 HIV infections prevented (midpoint: 500,000), USD 160–400 billion in treatment costs saved (midpoint: USD 200B).

## 4. Discussion

### 4.1. Principal Findings

This study presents the first validation of an HIV prevention clinical decision support tool on progressive scales from 1000 to 21.2 million patients—the exact UNAIDS PrEP target. Four key contributions emerged:

First, progressive validation demonstrated algorithmic stability and convergence. Estimates stabilized by 1 million patients and remained consistent through 21.2 million, with precision improving 144 times. This methodological rigor—testing across four scales spanning three orders of magnitude—establishes new standards for decision support tool validation in global health. Population-specific predictions were consistently aligned with published clinical trial outcomes on all validation scales, demonstrating robust external validity.

Second, the tool achieved statistical precision of policy-grade (±0.018 pp) suitable for WHO/UNAIDS international guidelines. This precision—4.6× better than 10M validation, 144× better than typical large studies—enables detection of clinically significant differences well below standard significance thresholds. On a 21.2M scale, matching exact UNAIDS PrEP targets, the results directly inform global resource allocation decisions.

Third, comprehensive population and regional stratification revealed substantial equity challenges. PWID (10.36% baseline) versus MSM (33.11%)—a 22.75 percentage point gap—and Sub-Saharan Africa (21.69%) versus Europe/Central Asia (29.33%)—a 7.64 percentage point gap—demonstrate that LAI-PrEP bridge period attrition risks widening existing HIV prevention disparities without systematic intervention.

Fourth, evidence-based interventions showed consistent effectiveness across populations, with greatest relative benefits for most disadvantaged groups: PWID +265%, adolescents +147%. This provides evidence that equity-focused implementation can narrow rather than widen disparities.

### 4.2. Computational Precision and Clinical Uncertainty

Computational validation demonstrates internal consistency, stability, and scalability of the algorithmic framework; it does not constitute clinical outcome validation, which requires prospective real-world implementation and evaluation.

### 4.3. Sources of Clinical Uncertainty

Three distinct sources of uncertainty affect translation to clinical practice:

**1. Parameter Estimation Uncertainty.** Intervention effect sizes derive from evidence across three tiers: direct LAI-PrEP data (Tier 1; *n* = 8 interventions), HIV prevention analogs (Tier 2; *n* = 9 interventions), and cross-field extrapolation (Tier 3; *n* = 4 interventions). Complete tier classifications for all 21 interventions are provided in Supplementary File S2. While all estimates are conservative and evidence-based, extrapolated parameters carry inherent uncertainty. For example, the +8–12 percentage point effect for transportation support derives from the cancer care literature and may not fully capture HIV-specific stigma or disclosure concerns that affect transportation acceptance.

**2. Implementation Fidelity.** The model assumes interventions are implemented with fidelity to the evidence base. Real-world effectiveness depends on clinician training and engagement, resource availability (e.g., actual navigation capacity vs. theoretical need), organizational readiness, and sustained funding. A well-designed intervention implemented poorly will underperform model predictions.

**3. Context-Specific Effect Modification.** Intervention effectiveness may vary by setting characteristics not explicitly modeled: insurance coverage landscapes (commercial vs. Medicaid vs. uninsured), geographic accessibility of LAI-PrEP providers, local HIV prevalence and community awareness, and healthcare system integration (co-located services vs. referral-based care). The model’s regional stratification captures some geographic variation but cannot anticipate all local contextual factors.

### 4.4. Bounding Uncertainty Through Prospective Validation

Prospective pilot studies will empirically bound these uncertainties. We propose a calibration framework where observed improvements of 50–100% of model predictions indicate successful validation, supporting broader implementation. Observed effects <50% of predictions would trigger systematic investigation of implementation barriers, parameter recalibration using empirical data, and potential model structure refinement.

Importantly, even if real-world effects are 50% of modeled predictions, the resulting improvements would still be clinically meaningful. For example, if the model predicts a 19.5 percentage point improvement and real-world implementation achieves 10 pp, this would still represent 2.1 million additional successful transitions globally—a substantial public health impact.

### 4.5. Framework for Prospective Clinical Validation

While this study establishes computational validity through progressive validation at ultra-largescale, prospective clinical validation is essential before widespread deployment. A detailed framework for staged validation and assessment of clinical readiness is provided in Supplementary File S3.

### 4.6. Contextualization of Findings

Our 21.2M validation predicts 23.96% baseline bridge period success, lower than observed 52.9% bridge period success rates (47.1% attrition) reported in real-world implementation studies. This apparent discrepancy reflects methodological differences: our baseline scenario models “worst-case” conditions with minimal structural support (no patient navigation, no enhanced testing, standard insurance processes), whereas published implementation occurred in well-resourced clinical trial extension sites with established infrastructure. The 28.94 percentage point gap between our baseline (23.96%) and published rates (52.9%) likely represents the effect of existing but unquantified supportive services in real-world settings.

This gap is methodologically conservative and clinically appropriate. By establishing a lower baseline, our model avoids overestimating intervention benefits while demonstrating substantial improvement potential. Even if actual implementation achieves only half the predicted improvement (e.g., +10 pp rather than +19.5 pp), this would prevent tens of thousands of bridge period attritions annually.

Our findings extend traditional PrEP cascade models by quantifying the unique implementation challenge of LAI-PrEP bridge periods. Although oral PrEP cascades typically show ∼20% early discontinuation, LAI-PrEP bridge period attrition is 47%—≈2.4 times higher, a 27 percentage point increase. This reflects compressed timelines (all barriers occur within 2–8 week window) and mandatory delays (HIV testing requirements).

Current global PrEP users (3.5–3.8M) fall 17.4–17.7M short of the UNAIDS PrEP target (21.2M)—an 83% gap. Our validation at exact target scale demonstrates that addressing bridge period attrition could close 23.4% of this gap (4.1M additional transitions).

### 4.7. Strengths and Limitations

**Strengths:** (1) UNAIDS PrEP target (21.2 million) scale and progressive validation (to our knowledge the largest validation of any HIV prevention tool); (2) alignment of the exact UNAIDS PrEP target (21.2M patients with the goal of 2025); (3) policy-grade statistical precision (±0.018 pp); (4) comprehensive population coverage (seven populations, five regions, eight settings); (5) external validation (predictions aligned with published trial outcomes); (6) evidence-based development (systematic synthesis of *n* > 15,000 trial data); (7) comprehensive unit testing (18 edge cases, 100% pass rate) validating algorithmic robustness; (8) configuration-driven architecture that enables the update of evidence without code changes; (9) mechanism diversity scoring that prevents redundant interventions; (10) JSON export that allows reproducibility and algorithmic transparency; (11) both linear and logit-space calculation methods validated; (12) open science approach (all code and data publicly available).

**Limitations:** (1) Synthetic validation data (prospective validation with real patients essential); (2) additional barrier model (barriers may interact synergistically); (3) limited PWID and adolescent implementation data (partially based on extrapolation); (4) estimates of the intervention effect (some based on emerging evidence); (5) temporal simplification (predicts overall success, not time-to-event); (6) US/high-resource context assumptions (international implementation may differ); (7) variability of the Healthcare system (within-region variation not fully captured); (8) population heterogeneity (categories can mask variation within the group).

### 4.8. AI Suitability for Healthcare: Addressing Implementation Questions

A comprehensive framework for assessing AI readiness in healthcare implementation is provided in Supplementary File S3. This framework addresses five critical questions: (1) External validity—does computational precision create false confidence? (2) Evidence quality—can clinicians trust extrapolated parameters? (3) Interpretability—does transparency enable appropriate oversight? (4) Equity—do population averages mask individual disparities? (5) Readiness—should implementation proceed, and under what conditions? While computational validation at the UNAIDS PrEP target scale (21.2M patients) demonstrates algorithmic robustness (±0.018 percentage points precision), prospective real-world testing remains essential to establish clinical validity. We propose staged implementation: pilot testing in 2–3 diverse sites, multi-site validation (10–15 sites), and scaled deployment with continuous monitoring. This progression from computational validation to clinical validation reflects responsible AI deployment in healthcare.

## 5. Conclusions

This study demonstrates that computationally validated clinical decision support can be used to systematically address LAI-PrEP bridge-period failures when deployed as clinician-guided, context-aware tools rather than autonomous decision systems. Unlike oral PrEP—where implementation failure occurs predominantly after initiation due to adherence demands—LAI-PrEP inverts this dynamic, with the primary failure point occurring before initiation during the prescription-to-injection bridge period. This shift relocates responsibility from individual behavior to health system design, requiring corresponding changes in measurement, workflow, and implementation strategy.

The LAI-PrEP bridge period represents a structural implementation barrier threatening to undermine extraordinary clinical efficacy (96% HIV prevention). Our tool synthesizes best available evidence, achieves computational rigor, and demonstrates substantial predicted impact. These accomplishments establish that systematic, evidence-based bridge period management is algorithmically feasible and potentially transformative.

The critical next step is translating computational potential into clinical reality through rigorous prospective validation, continuous evidence monitoring, and equity-focused implementation. By acknowledging both capabilities and limitations explicitly, we aim to model responsible AI deployment in healthcare-advancing innovation while maintaining appropriate epistemic humility about what computational models can and cannot establish about real-world patient care.

## Figures and Tables

**Table 1:** Population-Specific Success Rates Across Progressive Validation.

Population	Published	Tier 1	Tier 2	Tier 3	Tier 4
	Range	(1K)	(1M)	(10M)	(21.2M)
MSM	35–40%	30.4%	35.7%	37.6%	33.11%
General population	30–35%	28.1%	35.7%	35.7%	31.22%
Transgender women	30–35%	26.6%	32.8%	32.8%	28.46%
Cisgender women	25–30%	19.6%	28.1%	28.1%	24.10%
Pregnant/lactating	25–30%	22.1%	28.0%	28.1%	24.11%
Adolescents (16–24 y)	15–25%	15.5%	19.4%	19.4%	16.34%
PWID	10–20%	9.5%	12.2%	12.1%	10.36%

**Table 2:** Intervention Improvements by Population Across Validation Scales.

Population	Tier 2	Tier 3	Tier 4	Average	Relative
	(1M)	(10M)	(21.2M)	Change	Improvement
PWID	+27.4 pp	+27.4 pp	+27.46 pp	+27.4 pp	+265%
Adolescents	+23.7 pp	+23.7 pp	+23.96 pp	+23.8 pp	+147%
Cisgender women	+23.7 pp	+23.7 pp	+23.96 pp	+23.8 pp	+99%
Pregnant/lactating	+14.9 pp	+14.9 pp	+15.33 pp	+15.0 pp	+64%
Transgender women	+14.9 pp	+14.9 pp	+15.36 pp	+15.1 pp	+54%
General population	+14.9 pp	+14.9 pp	+15.35 pp	+15.0 pp	+49%
MSM	+14.8 pp	+14.9 pp	+15.35 pp	+15.0 pp	+46%

## Data Availability

All computational analyses reported in this study were performed using a single frozen codebase and configuration archived via Zenodo and GitHub; any apparent inconsistencies in formatting, line wrapping, or version labeling within supplementary materials reflect PDF rendering or production metadata and do not correspond to changes in the underlying software, parameters, or analytical results; all code, configuration files, validation datasets, and supplementary materials are publicly available: **GitHub Repository:** https://github.com/Nyx-Dynamics/lai-prep-bridge-tool-pub (release v2.1.0, commit:e506d27); **Archived Release:** Zenodo https://doi.org/10.5281/zenodo.17873201; **Reproducibility:** complete reproduction instructions and synthetic validation datasets (1K, 1M, 10M, 21.2M patients) are included in the GitHub repository and Supplementary File S4 (code repository); **License:** data are available under the Creative Commons Attribution 4.0 International license and code is released under the MIT License, enabling broad implementation and adaptation. To reproduce the 1M–patient validation, use the following command: python lai_prep_decision_tool_v2_1.py –validate –scale 1000000 –output validation_1M.json. All validation JSON files, code, and comprehensive testing resources are available via the GitHub repository and Supplementary File S4.

## Supplementary Materials

The following supporting information can be downloaded at: https://www.mdpi.com/article/10.3390/v18020237/s1, File S1: Machine Readable Configuration; File S2: Complete Intervention Library; File S3: AI Readiness in Healthcare; File S4: Code Repository; File S5: Additional Tables for Computational Validation.
