# Supplementary material for: Computational Validation of a Clinical Decision Support Algorithm for LAI-PrEP Bridge Period Navigation at UNAIDS PrEP Target Scale (21.2 Million Individuals)
Source: Viruses. 2026 Feb 13;18(2):237. doi: 10.3390/v18020237 (PMC12945109; doi:10.3390/v18020237)
Supplement: Supplementary file 1 [file viruses-18-00237-s001.zip › viruses-4063895-S1-final.pdf]

Supplementary Materials: COMPUTATIONAL VALIDATION OF A CLINICAL DECISION SUPPORT ALGORITHM FOR LAI–BRIDGE PERIOD NAVIGATION AT UNAIDS PREP TARGET SCALE (21.2 MILLION INDIVIDUALS)

Supplementary File S1

Machine Readable Configuration

Version 2.2 | December 2025 | Corresponds to configuration v3.1.0

Zenodo DOI:https://zenodo.org/record/17873201

Corresponding manuscript: Demidont, A.C. (2025). Validation of a Clinical Decision Support Algorithm for LAI-PrEP Bridge Period Navigation at UNAIDS PrEP Target Scale (21.2M Individuals). *Viruses*.

This supplementary file provides the machine-readable configuration files and parameter specifications that drive the LAI-PrEP bridge period decision support algorithm. These files enable reproducibility, external validation, and adaptation of the model to different clinical contexts and populations.

S1.1 Configuration File Structure

The algorithm implements a configuration-driven architecture using JavaScript Object Notation (JSON) format. This approach separates algorithmic logic from clinical parameters, enabling:

- Rapid updates as new evidence emerges without modifying code
- Version control and comparison across parameter sets
- External audit and sensitivity analysis
- Local adaptation based on implementation experience
- Prospective validation with alternative assumptions

S1.2 Core Configuration Parameters

The configuration file specifies:

Population Baseline Rates Success rates by population, source evidence, and confidence tier (Tier 1: LAI-PrEP data; Tier 2: Oral PrEP data; Tier 3: Cross-therapeutic extrapolation)

Structural Barriers Barriers to bridge period completion (n=21), with impact weights as percentage-point reductions in success probability, evidence sources, and implementation indicators

Evidence-Based Interventions Interventions to address barriers (n=21), effect sizes, evidence levels (High/Moderate/Emerging), mechanisms of action, overlap penalties, and implementation complexity ratings

Modeling Parameters Diminishing returns factors (default 70%), barrier combination method (multiplicative default), overlap penalty structure, and validation checksums

Version Control Configuration version number, timestamp, change log, and backward compatibility settings

S1.3 Population-Specific Baseline Rates

Table S1. Population-Specific Bridge Period Success Rates (Baseline)

Population	Baseline Success (%)	Source Tier	Evidence Reference
Cisgender men who have sex with men (MSM)	52.9	Tier 1	HPTN 083, PURPOSE-1 [1,2]
Transgender women	38.4	Tier 2	PURPOSE-2 trial analysis, HPTn083 [1,2]
People who inject drugs (PWID)	24.6	Tier 3	Oral PrEP cascade data [3]
Adolescents (13–24 years)	28.3	Tier 1 and 3	HVTN 702 analog, expert consensus[4,5]
Sub-Saharan Africa (general Cisgender women population)	31.2	Tier 2	HPTN 084 implementation data [4,6]
North America/Europe (high-income)	48.7	Tier 1	Trial data + implementation [7,8]

[1]

S1.4 Structural Barriers (n=21) with Impact Weights

Table S1.1 in the complete configuration file documents all 21 barriers with:

- Barrier name and category (financial, logistic, clinical, educational, social)
- Impact weight as percentage-point reduction in baseline success
- Evidence source (published literature, qualitative studies, expert consensus)
- Implementation indicators (how to identify barrier presence)
- Mitigation requirements for each barrier

S1.5 Evidence-Based Interventions (n=21) with Effect Sizes

Table S1.2 documents all 21 interventions with:

[1] Baseline rates for Sub-Saharan Africa derive primarily from cisgender women trials (HPTN 084, PURPOSE-1), the only populations with robust LAI-PrEP-specific data in the region. Notably, cisgender women demonstrate among the lowest oral PrEP cascade completion rates globally despite strong trial outcomes, underscoring the critical gap between efficacy and real-world implementation that this tool addresses. Generalizability to MSM, transgender women, PWID, and adolescents in SSA requires population-specific validation as implementation data emerge.

- Intervention name and mechanism of action
- Effect size (percentage-point improvement in bridge period success)
- Evidence level (High: RCT or robust implementation data; Moderate: quasi-experimental or observational with controls; Emerging: pilot or expert consensus)
- Published references for effect size derivation
- Complexity rating (Low/Medium/High) for implementation
- Overlap penalties with other interventions (to account for synergistic effects and ceiling constraints)

[2]

S1.6 Validation Checksums and Data Integrity

All configuration files include SHA-256 checksums for each parameter section. These enable:

- Detection of unintended modifications
- Verification that external users are working with identical configuration
- Retrospective confirmation of which version was used for published analyses

S1.7 Configuration Version History

The configuration file maintains a complete version history:

v1.0.0 Initial configuration (used for primary validation analysis)

v2.0.0 Updated intervention effect sizes based on 2024 real-world implementation data (HPTN 083-02, Trio Health Cohort)

v2.1.0 (Current) Includes new published evidence on transportation barriers and financial navigation support

Each version includes timestamp, substantive changes, and backward compatibility notes.

S1.8 JSON Configuration File Example Structure

The complete machine-readable configuration file follows this structure:

Listing 1: Configuration File Structure Example

```
{
  "metadata": {
    "version": "2.1.0",
    "date": "2025-12-12",
    "algorithm_name": "LAI-PrEP Bridge Period Decision Support
       ",
    "doi": "10.5281/zenodo.17873201"
  },
  "populations": [
    {
      "name": "MSM",
      "baseline_success_rate": 0.529,
      "source_tier": "Tier 1",
      "evidence_reference": "HPTN_083_2024"
    }
```

[2] SSA estimates extrapolate from cisgender women trial data (HPTN 084, PURPOSE-1); other populations in the region lack direct LAI-PrEP implementation evidence.

```
  ],
  "barriers": [
    {
      "id": "B001",
      "name": "Financial barriers",
      "impact_weight": -0.12,
      "evidence_level": "High",
      "mechanism": "Insurance gaps, copayment burdens"
    }
  ],
  "interventions": [
    {
      "id": "I001",
      "name": "Financial navigation support",
      "effect_size": 0.15,
      "evidence_level": "High",
      "complexity": "Medium"
    }
  ],
  "modeling_parameters": {
    "diminishing_returns_factor": 0.70,
    "barrier_combination_method": "multiplicative",
    "max_attrition_ceiling": 0.95
  }
}
```


S1.9 Accessing Configuration Files

Complete machine-readable configuration files are available at:

- GitHub Repository: `https://github.com/nyx-dynamics/lai-prep-decision-support`
- Zenodo Archive: DOI `https://doi.org/10.5281/zenodo.17873201`
- MDPI Supplementary Materials: Attached configuration files with checksums

S1.10 Customization and Adaptation

Sites implementing this tool can adapt configurations by:

1. Modifying population baseline rates based on local epidemiology
2. Adjusting barrier impact weights using local implementation data
3. Updating intervention effect sizes as new evidence emerges
4. Maintaining version control to document all modifications
5. Validating checksums before deployment to ensure data integrity

All changes should be documented with timestamp, rationale, and evidence source.

S1.11 Validation and Testing

All configuration parameters undergo automated validation:

- Parameter bounds checking (probabilities constrained to 0–1 range)
- Mathematical consistency (no circular dependencies, invalid diminishing returns)
- Evidence source verification (all cited references available)
- Sensitivity analysis across parameter ranges

- Robustness testing with alternative assumptions

S1.12 Documentation and Audit Trail

Each configuration file includes:

- Complete data dictionary with definitions
- Source documentation for every parameter value
- Confidence intervals or uncertainty ranges where available
- Date of last validation
- Contacts for questions about specific parameters

References

Reference: A.C Demidont, DO(2025). Computational Validation of a Clinical Decision Support Algorithm for Long-Acting Injectable PrEP Bridge Period Navigation at UNAIDS Global Target Scale. *Viruses*

==

References, variant A: external bibliography

1. Landovitz, R.J.; Donnell, D.; Clement, M.E.; Hanscom, B.; Cottle, L.; Coelho, L.; et al. Cabotegravir for HIV prevention in cisgender men and transgender women. *New England Journal of Medicine* **2021**, *385*, 595–608. https://doi.org/10.1056/NEJMoa2101016.
2. Kelley, C.F.; Acevedo-Quiñones, M.; Agwu, A.L.; et al. Twice-yearly lenacapavir for HIV prevention in men and gender-diverse persons. *New England Journal of Medicine* **2025**, *392*, 1261–1276. https://doi.org/10.1056/NEJMoa2411858.
3. Mistler, C.B.; Copenhaver, M.M.; Shrestha, R. The pre-exposure prophylaxis (PrEP) care cascade in people who inject drugs: A systematic review. *AIDS and Behavior* **2021**, *25*, 1490–1506. https://doi.org/10.1007/s10461-020-02988-x.
4. Bekker, L.G.; Das, M.; Abdool Karim, Q.; et al. Twice-yearly lenacapavir or daily F/TAF for HIV prevention in cisgender women. *New England Journal of Medicine* **2024**, *391*, 1179–1192. https://doi.org/10.1056/NEJMoa2407001.
5. Hosek, S.G.; Landovitz, R.J.; Kapogiannis, B.; et al. Safety and feasibility of antiretroviral preexposure prophylaxis for adolescent men who have sex with men. *JAMA Pediatrics* **2017**, *171*, 1063. https://doi.org/10.1001/jamapediatrics.2017.2007.
6. Delany-Moretlwe, S.; Hughes, J.P.; Bock, P.; Ouma, S.G.; Hunidzarira, P.; et al. Cabotegravir for the prevention of HIV-1 in women: Results from HPTN 084. *The Lancet* **2022**, *399*, 1779–1789. https://doi.org/10.1016/S0140-6736(22)00538-4.
7. Altamirano, J.A.; Shukla, P.; Barnett, S.K. 1531. Early real-world experience of long-acting cabotegravir (CAB) for HIV pre-exposure prophylaxis (PrEP) in a large community-based clinic network (CAN Community Health): Utilization and PrEP persistence. *Open Forum Infectious Diseases* **2023**, *10*, ofad500.1366. https://doi.org/10.1093/ofid/ofad500.1366.
8. Ramgopal, M.; Brown, C.A.; Frick, A.; et al. Real-world use of cabotegravir long-acting PrEP: Trio Health cohort. *Open Forum Infectious Diseases* **2025**, *12*, ofae631.157. https://doi.org/10.1093/ofid/ofae631.157.
