# Supplementary material for: Computational Validation of a Clinical Decision Support Algorithm for LAI-PrEP Bridge Period Navigation at UNAIDS PrEP Target Scale (21.2 Million Individuals)
Source: Viruses. 2026 Feb 13;18(2):237. doi: 10.3390/v18020237 (PMC12945109; doi:10.3390/v18020237)
Supplement: Supplementary file 1 [file viruses-18-00237-s001.zip › viruses-4063895-S2-final.pdf]

Supplementary Materials: COMPUTATIONAL VALIDATION OF A CLINICAL DECISION SUPPORT ALGORITHM FOR LAI–BRIDGE PERIOD NAVIGATION AT UNAIDS PREP TARGET SCALE (21.2 MILLION INDIVIDUALS)

Supplementary File S2

Complete Intervention Library

Evidence-Based Strategies for LAI-PrEP Bridge Period Navigation

21 Interventions with Evidence Sources, Effect Sizes, and Implementation Guidance

Version 2.2 | December 2025 | Corresponds to configuration v3.1.0

Zenodo DOI:https://zenodo.org/record/17873201

Corresponding manuscript: Demidont, A.C. (2025). Validation of a Clinical Decision Support Algorithm for LAI-PrEP Bridge Period Navigation at UNAIDS PrEP Target Scale (21.2M Individuals). *Viruses*.

Purpose

This comprehensive intervention library synthesizes evidence from LAI-PrEP clinical trials (HPTN 083, HPTN 084, PURPOSE-1/2)[1–4], implementation studies, and analogous healthcare interventions (cancer screening navigation, oral PrEP cascades, HIV care continuum). Each intervention includes:

- **Mechanism classification** for diversity-aware selection
- **Effect size estimates** from published literature
- **Evidence strength ratings** (Strong/Moderate/Emerging)
- **Implementation complexity** assessment
- **Target populations** and barrier specificity
- **Mechanism tags** for algorithm diversity scoring

The mechanism diversity scoring prevents redundant recommendations by selecting interventions with complementary mechanisms of action.

Table S1. Complete Intervention Library: Evidence-Based Strategies for LAI-PrEP Bridge Period Navigation (n=21 interventions). All interventions include documented evidence sources, estimated effect sizes, implementation complexity, and mechanism classifications enabling diversity-aware selection.

Intervention	Description	Mechanisms & Tags	Effect Size	Evidence Level & Source	Implementation Complexity
**ELIMINATE THE BRIDGE PERIOD**					

Continued on next page

Table S1 – *Continued from previous page*

Intervention	Description	Mechanisms & Tags	Effect Size	Evidence Level & Source	Implementation Complexity
Oral-to-Injectable Same-Day Switching	Eliminate mandatory re-testing delay for patients with recent negative HIV test on oral PrEP	*eliminate_bridge* (primary), *structural_support* (secondary)	+35% absolute (88–90% vs 53% baseline)	**Strong**: CAN study [5], Ryan White LA-ART data [6]	Low (policy change)
**COMPRESS THE BRIDGE PERIOD**					
HIV-1 RNA Testing	Reduce mandatory window period from 33–45 days to 10–14 days post-exposure	*compress_bridge* (primary)	+15–20% [7–9]	**Moderate**: WHO 2025 guidelines [10], CDC recommendations [7,8]; HPTN 083 Screening Analysis [11]	Medium (lab infras-tructure)
Rapid Laboratory Turnaround (24–48h)	Accelerate test-to-result time, reducing total bridge duration by 3–5 days	*compress_bridge* (primary), *structural_support* (secondary)	+10–15%	**Moderate**: Lab optimization studies [7–9]	Medium (system redesign)
Point-of-Care HIV Testing	Enable same-day testing at injection visit, eliminating separate testing appointment	*compress_bridge* (primary), *remove_barriers* [8,9] (secondary)	+8–12%	**Emerging**: FDA-approved Ag/Ab POC available ; RNA POC limited	High (technol-ogy adoption)[12]
**NAVIGATE THE BRIDGE PERIOD**					
Dedicated Patient Navigation	Trained navigator coordinates appointments, insurance, transportation, addresses information gaps	*navigate_bridge* (primary), *structural_support, clinical_support* (secondary)	+12–20% (1.5–2× improve-ment)	**Strong**: SF PrEP navigation [13] HR 1.5; Cancer care meta-analysis [14]	Medium (staffing)
Peer Navigation	Peer navigators with lived experience provide culturally-congruent support, reduce mistrust	*navigate_bridge* (primary), *clinical_support* (secondary)	+15–20% for key popula-tions	**Moderate**: HIV care cascade peer navigation [15,16]; greater effect than non-peer [17–19]	Medium (recruit-ment, training)
SMS/Text Message Reminders	Automated appointment reminders, adherence support, reduce no-shows by 20–30%	*navigate_bridge* (primary)	+10–15%	**Strong**: Meta-analyses across healthcare conditions [20,21]	Low (existing plat-forms)
Population-Tailored Navigation	Adolescent-specific (address autonomy, parental consent), PWID-specific (harm reduction integration), etc.	*navigate_bridge, clinical_support, system_level*	+20–30% for highest-barrier groups	**Moderate**: Population-specific literature [18,19]	Medium-High (special-ization)
**REMOVE FINANCIAL & LOGISTICAL BARRIERS**					
Transportation Support	Ride-share vouchers, transit passes, mileage reimbursement for multiple appointments	*remove_barriers* (primary)	+10–15% (high impact for women, rural)	**Moderate**: Cancer care transportation studies [14]; PrEP barrier literature [22]	Low-Medium (voucher systems)
Childcare Assistance	On-site childcare or vouchers enabling appointment attendance for parents/caregivers	*remove_barriers* (primary)	+8–12% (concen-trated among care-givers)	**Emerging**: Family planning service parallels [23]	Medium (facility/-partner-ships)

Continued on next page

Table S1 – *Continued from previous page*

Intervention	Description	Mechanisms & Tags	Effect Size	Evidence Level & Source	Implementation Complexity
Mobile Delivery Services	Home or community-based injection services, eliminate clinic visit barriers	*remove_barriers, system_level*	+15–25%	**Moderate**: Novel PrEP delivery platforms (mobile/community) [16]; LAI-CAB delivery pathway considerations [24]; WHO 2025 guidance [10]	High (mobile units, staffing)
Bundled Payment Models	Single authorization covers all bridge period services, streamlines multi-visit approval	*structural_support, system_level*	+12–18%	**Emerging**: Episode-based payment theory [22]	High (payer negotiation)
Accelerated Insurance Authorization	Priority review pathway, reduce 7–14 day delays affecting 30–40% of patients	*structural_support* (primary)	+12–15%	**Emerging**: Health policy literature on prior authorization [22]	Medium (payer partnerships)
**ADDRESS CLINICAL & INTERPERSONAL BARRIERS**					
Medical Mistrust Intervention	Community health worker support, cultural concordance, address historical trauma	*clinical_support* (primary)	+8–12%	**Moderate**: Patient navigation for marginalized populations [25,26]	Medium (CHW training)
Anti-Discrimination Protocols	LGBTQ+-affirming care, staff training, visible inclusivity signals	*clinical_support* (primary)	+10–15% for SGM populations	**Moderate**: Sexual and gender minority healthcare literature [27,28]	Low-Medium (training)
Confidentiality Protections	Youth-friendly services, anonymous scheduling, privacy-preserving systems	*clinical_support* (primary)	+8–12% for adolescents	**Moderate**: Adolescent PrEP literature [29]	Medium (system redesign)
Language-Concordant Services	Professional interpretation, translated materials, multilingual staff	*clinical_support, remove_barriers*	+10–12% for LEP populations	**Moderate**: Healthcare language access studies [30]	Medium (interpreter services)
**SYSTEM-LEVEL REDESIGN**					
Telemedicine Integration	Virtual visits for counseling/follow-up, reduce in-person visits from 3 to 2	*navigate_bridge, remove_barriers, system_level*	+10–15%	**Moderate**: Telehealth innovations for PrEP delivery [31]; differentiated delivery platforms including telemedicine [16]; injectable CAB implementation considerations [24]	Medium (technology platform)
Pharmacist-Led Prescribing	Expand prescriber pool 5–10×, reduce provider appointment barriers	*system_level, structural_support*	+15–20%	**Moderate**: Pharmacy-led PrEP delivery platforms [16]; task shifting frameworks [32]; implementation strategies [17]	High (regulatory change)
Harm Reduction Integration (PWID)	Co-locate LAI-PrEP with syringe services, reduce stigma/criminalization fears	*system_level, clinical_support*	+25–35% for PWID (10% baseline → 35–45%)	**Moderate**: SSP-integrated HIV services [27]	Medium-High (service integration)

Continued on next page

Table S1 – *Continued from previous page*

Intervention	Description	Mechanisms & Tags	Effect Size	Evidence Level & Source	Implementation Complexity
Community-Based Delivery	Deliver services in community settings vs. clinical facilities, address medical mistrust	*system_level, clinical_support, remove_barriers*	+15–25% in under-resourced settings	**Moderate**: Community/mobile and peer-supported platforms [16]; LAI-CAB implementation considerations [24]; WHO 2025 guidance [10]; disparity reduction frameworks [33]	High (community partnerships)

Methodological Notes

Effect Size Estimation and Evidence Quality

All effect sizes in this library are derived from:

1. **Direct LAI-PrEP implementation data** (when available): CAN Community Health Network study
2. **Oral PrEP RWE implementation metadata for extrapolation** (with caution): Effect sizes from oral PrEP navigation interventions, adjusted for LAI-specific barriers
3. **Analogous healthcare interventions**: Cancer screening navigation, HIV treatment cascade, maternal health navigation programs with similar structural barriers

Evidence strength ratings:

- **Strong**: Multiple studies, meta-analyses, or large implementation cohorts with consistent findings
- **Moderate**: Limited studies, single large cohort, or extrapolation from closely analogous settings
- **Emerging**: Theoretical rationale, pilot data, or extrapolation from less directly comparable interventions

Intervention Effect Calculation: Two-Stage Model

The algorithm uses two distinct parameters for combined intervention effects:

Stage 1: Diminishing Returns Factor ($\alpha = 0.70$)

Individual intervention effects (after mechanism overlap penalties) are summed, then multiplied by diminishing returns factor:

$$\Delta\text{Success}_{intermediate} = 0.70 \times \sum_{i=1}^{n} e_i \tag{1}$$

where e_i is the adjusted effect of intervention i.

Rationale: Multi-component healthcare interventions typically yield 60–80% of their theoretical additive effect due to:

- Overlapping mechanisms (e.g., both navigation and transportation help with appointment attendance)
- Patient saturation effects (limited capacity to participate in multiple simultaneous interventions)
- Irreducible failure modes (e.g., patients who move out of state during bridge period)

Example: Three interventions with adjusted effects +8%, +10%, +12%:

- **Naive additive prediction**: 8 + 10 + 12 = 30% improvement
- **Realistic combined effect**: 0.70 × 30 = 21% improvement

Stage 2: Absolute Success Rate Ceiling (max = 0.95)

The final success rate (baseline + Stage 1 improvement) is capped at maximum absolute success rate of 95%:

$$\text{Success}_{final} = \min(\text{Success}_{baseline} + \Delta\text{Success}_{intermediate}, 0.95) \quad (2)$$

Important Note: This ceiling represents maximum *absolute* success rate, not maximum improvement. The maximum possible improvement therefore varies by baseline:

Baseline Success	Max Final Success	Max Improvement
10%	95%	85 percentage points
25%	95%	70 percentage points
50%	95%	45 percentage points
75%	95%	20 percentage points
90%	95%	5 percentage points

Rationale: Even with optimal intervention bundles, some attrition is unavoidable due to:

- Patient relocation outside service area
- Insurance changes or loss of coverage
- Personal decisions to discontinue PrEP
- Unforeseeable life events (hospitalization, family emergencies)

The 95% ceiling reflects clinical reality that some small proportion of patients will not successfully complete bridge period regardless of interventions.

Validation data: In 21.2 million patient validation, average success with interventions was 43.5%, well below the 95% ceiling, confirming the ceiling is appropriate and not artificially constraining predictions.

Configuration note: Both parameters are externally configurable in the JSON file (configuration v3.1.0):

- `intervention_diminishing_returns_factor: 0.70`
- `max_success_rate_with_interventions: 0.95`

Sensitivity analysis shows results are robust to variations in α from 0.60 to 0.80 ($\pm$2.5 percentage points).

Mechanism Classification System

The mechanism diversity scoring uses six categories to prevent redundant recommendations:

1. **eliminate_bridge:** Interventions that completely remove the bridge period (e.g., same-day switching for oral PrEP patients with recent HIV test)[5,6]
2. **compress_bridge:** Interventions that shorten bridge duration without eliminating it (e.g., RNA testing reducing window period, rapid lab turnaround)[7–12]
3. **navigate_bridge:** Interventions that guide patients through existing bridge period (e.g., patient navigation, peer navigation, text reminders)
4. **remove_barriers:** Interventions that eliminate specific structural obstacles (e.g., transportation support, childcare, mobile delivery)
5. **clinical_support:** Interventions addressing interpersonal and clinical barriers (e.g., medical mistrust interventions, cultural concordance, confidentiality protections)
6. **structural_support:** Interventions targeting systemic/administrative barriers (e.g., insurance authorization, bundled payments, rapid lab processing)

7. **system_level:** Interventions requiring fundamental healthcare delivery redesign (e.g., pharmacist prescribing, harm reduction integration, community-based delivery)

[10]

Mechanism Overlap Penalty

When selecting multiple interventions, the algorithm applies a 10% penalty for each shared mechanism tag:

$$\text{adjusted\_effect} = \text{base\_effect} \times (1 - 0.10 \times k) \tag{3}$$

where k is the number of mechanism tags shared with already-selected interventions.
Example:

1. **First intervention** (Patient Navigation): *navigate_bridge, structural_support* → +12% (no penalty, first selection)
2. **Second intervention** (Peer Navigation): *navigate_bridge* → +10% × (1 - 0.10×1) = +9% (1 shared tag with #1)
3. **Third intervention** (Transportation Support): *remove_barriers* → +8% (no penalty, distinct mechanisms)
4. **Fourth intervention** (Insurance Navigation): *structural_support* → +10% × (1 - 0.10×1) = +9% (1 shared tag with #1)
5. **Fifth intervention** (Medical Mistrust): *clinical_support* → +12% (no penalty, distinct mechanisms)

Total: 12 + 9 + 8 + 9 + 12 = 50% (sum of adjusted effects)
After Stage 1 (α=0.70): 0.70 × 50 = 35% improvement
Final (Stage 2): If baseline = 24%, final = min(24+35, 95) = 59% success rate

This penalty ensures diverse approaches addressing complementary failure modes rather than redundant strategies.

Barrier Impact Calculation

Individual barrier impacts in this library reflect **marginal effects** assuming multiplicative combination (as specified in algorithm configuration). When multiple barriers are present:

$$P_{\text{attrition}} = 1 - \prod_{j=1}^{m} (1 - b_j) \tag{4}$$

where b_j is the impact of barrier j.

This multiplicative approach reflects that barriers often interact synergistically (e.g., transportation barriers are more severe when combined with childcare needs), and prevents mathematical impossibilities that can occur with simple addition.

Target Populations and Barrier Specificity

Universal interventions (applicable to all populations):

- Patient navigation
- SMS/text reminders
- Accelerated HIV testing
- Same-day switching (for oral PrEP patients)

Testing strategy note: HIV testing and diagnostic timing components (including RNA testing and point-of-care testing) are based on WHO and U.S. guidance and HPTN assay analyses [7–12]. **Population-tailored interventions:**

- **PWID:** Harm reduction integration, peer navigation, mobile delivery

- **Adolescents:** Youth-specific navigation, confidentiality protections, school-friendly scheduling
- **Cisgender women:** Transportation support, childcare assistance, community-based delivery
- **Transgender women:** Anti-discrimination protocols, peer navigation, affirming care training
- **Rural populations:** Mobile delivery, telemedicine, transportation support

Delivery model note: Differentiated delivery options (telemedicine, mobile/community delivery, pharmacy-led models, and task shifting) are supported by telehealth and platform reviews and implementation frameworks [10,16,24,31].

Barrier-specific interventions:

- Transportation barriers → Transportation support, mobile delivery, telemedicine
- Childcare barriers → Childcare assistance, extended hours, home delivery
- Insurance barriers → Expedited authorization, bundled payments
- Medical mistrust → CHW interventions, peer navigation, cultural concordance
- Scheduling conflicts → Extended hours, telemedicine, mobile delivery
- Privacy concerns → Confidentiality protections, community-based delivery

Evidence Gaps and Future Research

Distinction: Computational vs Clinical Validation

What has been validated computationally (at 21.2M patient scale):

- Algorithmic stability across scales (1K to 21.2M patients)
- Mathematical consistency of probability calculations
- Convergence of estimates with increasing sample size (95% CI: $\pm$0.018 percentage points at 21.2M)
- Sensitivity to parameter variations (α from 0.60 to 0.80: $\pm$2.5 points)
- Edge case handling (100% test pass rate on 18 edge cases)

What requires clinical validation (prospective implementation research):

1. **LAI-PrEP-specific effect sizes:** Most estimates extrapolate from oral PrEP or analogous interventions. Direct LAI-PrEP implementation trials needed.
2. **Synergistic interactions:** Current model assumes additive effects (with diminishing returns). Some intervention combinations may have multiplicative benefits.
3. **Optimal intervention bundle size:** At what point do additional interventions provide minimal incremental benefit?
4. **Population heterogeneity:** Effect sizes may vary substantially within broad categories.
5. **Cost-effectiveness ratios:** Which interventions provide best value for investment?
6. **Implementation fidelity:** Real-world effectiveness depends on intervention quality and protocol adherence.
7. **Sustainability:** Long-term maintenance of intervention programs beyond pilot funding.
8. **Regional adaptation:** How do effect sizes vary across healthcare systems and cultural contexts?

Critical Note on Computational Precision: The tool achieves exceptional computational precision ($\pm$0.018 percentage points at 21.2M scale), but this does NOT imply clinical certainty about parameters. Parameter uncertainty remains substantial pending prospective validation.

https://doi.org/10.3390/v18020237

Use in Clinical Decision Support Tool

This intervention library serves as the external configuration for the LAI-PrEP Bridge Decision Support Tool. The tool:

1. **Matches interventions to barriers:** Identifies patient-specific barriers and selects interventions targeting those barriers
2. **Applies mechanism diversity scoring:** Prevents recommending multiple interventions with redundant mechanisms (10% penalty per shared tag)
3. **Calculates combined effects:** Uses two-stage model (Stage 1: 70% diminishing returns; Stage 2: 95% absolute success ceiling)
4. **Prioritizes by effect size and evidence:** Ranks recommendations by expected impact and evidence strength
5. **Considers implementation complexity:** Flags high-complexity interventions requiring substantial resources
6. **Enables local adaptation:** Sites can modify effect sizes, add interventions, or disable unavailable strategies by editing the JSON configuration file

Continuous updates: As new evidence emerges from ongoing trials and real-world implementation, this library should be updated to reflect improved effect size estimates and evidence strength ratings. Version control and change logs maintained in GitHub repository.

Conclusion

This comprehensive intervention library represents the synthesis of best available evidence for LAI-PrEP bridge period navigation. The 21 interventions span six mechanism categories, three evidence strength levels, and three implementation complexity tiers, enabling flexible, evidence-based decision support tailored to patient populations, local resources, and specific barriers.

Implementation readiness: The tool is computationally validated at global scale (21.2M synthetic patients matching UNAIDS 2025 global targets) with policy-grade precision ($\pm$0.018 percentage points). Open-source code, comprehensive documentation, and externalized configuration enable rapid deployment.

Caveat: While computational validation demonstrates algorithmic precision, prospective validation with real patients in diverse settings is essential to validate effect size estimates, identify optimal intervention bundles, refine implementation strategies, and establish clinical utility.

Correspondence to main manuscript: A.C Demidont, DO (2025). Computational Validation of a Clinical Decision Support Algorithm for Long-Acting Injectable PrEP Bridge Period Navigation at UNAIDS Global Target Scale. *Viruses*.

Software repository: https://github.com/Nyx-Dynamics/LAI-PrEP-Bridge-Tool
Zenodo DOI: https://zenodo.org/record/17873201 (v3.1)
Configuration file: See Supplementary File S1 (JSON Configuration v3.1.0)
Code documentation: See Supplementary File S4 (Code & Data Repository)

Reference: A.C Demidont, DO(2025). Computational Validation of a Clinical Decision Support Algorithm for Long-Acting Injectable PrEP Bridge Period Navigation at UNAIDS Global Target Scale. *Viruses*

1. Landovitz, R.J.; Donnell, D.; Clement, M.E.; Hanscom, B.; Cottle, L.; Coelho, L.; et al. Cabotegravir for HIV prevention in cisgender men and transgender women. *New England Journal of Medicine* **2021**, *385*, 595–608. https://doi.org/10.1056/NEJMoa2101016.
2. Delany-Moretlwe, S.; Hughes, J.P.; Bock, P.; Ouma, S.G.; Hunidzarira, P.; et al. Cabotegravir for the prevention of HIV-1 in women: Results from HPTN 084. *The Lancet* **2022**, *399*, 1779–1789. https://doi.org/10.1016/S0140-6736(22)00538-4.
3. Bekker, L.G.; Das, M.; Abdool Karim, Q.; et al. Twice-yearly lenacapavir or daily F/TAF for HIV prevention in cisgender women. *New England Journal of Medicine* **2024**, *391*, 1179–1192. https://doi.org/10.1056/NEJMoa2407001.
4. Kelley, C.F.; Acevedo-Quiñones, M.; Agwu, A.L.; et al. Twice-yearly lenacapavir for HIV prevention in men and gender-diverse persons. *New England Journal of Medicine* **2025**, *392*, 1261–1276. https://doi.org/10.1056/NEJMoa2411858.
5. Ramgopal, M.; Brown, C.A.; Frick, A.; et al. Real-world use of cabotegravir long-acting PrEP: Trio Health cohort. *Open Forum Infectious Diseases* **2025**, *12*, ofae631.157. https://doi.org/10.1093/ofid/ofae631.157.
6. Haser, G.C.; Balter, L.; Gurley, S.; Thomas, M.; Murphy, T.; et al. Early implementation of long-acting injectable cabotegravir/rilpivirine at Ryan White clinics in the U.S. South. *AIDS Research and Human Retroviruses* **2024**, *40*, 690–700. https://doi.org/10.1089/AID.2024.0007.
7. Patel, R.R.; Hoover, K.W.; Lale, A.; et al. Clinical recommendation for the use of injectable lenacapavir as HIV preexposure prophylaxis—United States, 2025. *MMWR Morbidity and Mortality Weekly Report* **2025**, *74*, 541–549. https://doi.org/10.15585/mmwr.mm7435a1.
8. Centers for Disease Control and Prevention. US Public Health Service: Preexposure Prophylaxis for the Prevention of HIV Infection in the United States—2021 Update, 2021.
9. Tanner, M.R.; O'Shea, J.G.; Byrd, K.M.; Dumitru, G.G.; Le, J.N.; Lale, A.; Byrd, K.K.; Cholli, P.; Kamitani, E.; Zhu, W.; et al. Antiretroviral Postexposure Prophylaxis After Sexual, Injection Drug Use, or Other Nonoccupational Exposure to HIV — CDC Recommendations, United States, 2025. *MMWR. Recommendations and Reports* **2025**, *74*, 1–56. https://doi.org/10.15585/mmwr.rr7401a1.
10. World Health Organization. *Guidelines on Lenacapavir for HIV Prevention and Testing Strategies for Long-Acting Injectable PrEP*; World Health Organization: Geneva, Switzerland, 2025.
11. Eshleman, S.H.; Fogel, J.M.; Halvas, E.K.; Piwowar-Manning, E.; Marzinke, M.A.; Kofron, R.; Wang, Z.; Mellors, J.; McCauley, M.; Rinehart, A.R.; et al. HIV RNA Screening Reduces Integrase Strand Transfer Inhibitor Resistance Risk in Persons Receiving Long-Acting Cabotegravir for HIV Prevention. *The Journal of Infectious Diseases* **2022**, *226*, 2170–2180. https://doi.org/10.1093/infdis/jiac415.
12. Fogel, J.M.; Piwowar-Manning, E.; Moser, A.; Hill, T.; Ahmed, S.; Cummings, V.; Mostafa, H.H.; Wang, Z.; Jennings, A.; Gallardo-Cartagena, J.A.; et al. Evaluation of Xpert point-of-care assays for detection of HIV infection in persons using long-acting cabotegravir for pre-exposure prophylaxis. *Microbiology Spectrum* **2024**, *12*, e00307–24. https://doi.org/10.1128/spectrum.00307-24.
13. Spinelli, M.A.; Scott, H.M.; Vittinghoff, E.; et al. A panel management and patient navigation intervention is associated with earlier PrEP initiation in a safety-net primary care health system. *Journal of Acquired Immune Deficiency Syndromes* **2018**, *79*, 347–351. https://doi.org/10.1097/QAI.0000000000001801.
14. Starbird, L.E.; DiMaina, C.; Sun, C.A.; Han, H.R. A systematic review of interventions to minimize transportation barriers among people with chronic diseases. *Journal of Community Health* **2019**, *44*, 400–411. https://doi.org/10.1007/s10900-018-0572-3.
15. Shade, S.B.; Kirby, V.B.; Stephens, S.; Moran, L.; Charlebois, E.D.; Xavier, J.; Cajina, A.; Steward, W.T.; Myers, J.J. Outcomes and costs of publicly funded patient navigation interventions to enhance HIV care continuum outcomes in the United States: A before-and-after study. *PLOS Medicine* **2021**, *18*, e1003418. https://doi.org/10.1371/journal.pmed.1003418.
16. Rousseau, E.; Julies, R.F.; Madubela, N.; Kassim, S. Novel platforms for biomedical HIV prevention delivery to key populations. *Current HIV/AIDS Reports* **2021**, *18*, 500–507. https://doi.org/10.1007/s11904-021-00578-7.

17. Sullivan, P.S.; Mena, L.; Elopre, L.; Siegler, A.J. Implementation Strategies to Increase PrEP Uptake in the South. *Current HIV/AIDS Reports* **2019**, *16*, 259–269. https://doi.org/10.1007/s11904-019-00447-4.
18. Biello, K.B.; Bazzi, A.R.; Mimiaga, M.J.; et al. Perspectives on HIV pre-exposure prophylaxis (PrEP) utilization and related intervention needs among people who inject drugs. *Harm Reduction Journal* **2018**, *15*, 55. https://doi.org/10.1186/s12954-018-0263-5.
19. Mixson, L.S.; Zule, W.; Ruderman, S.A.; et al. Multiple injections per episode among people who inject drugs in rural U.S. communities. *International Journal of Drug Policy* **2025**, *143*, 104837. https://doi.org/10.1016/j.drugpo.2025.104837.
20. Nijhawan, A.E.; Metsch, L.R.; Zhang, S.; et al. Clinical and sociobehavioral prediction model of 30-day hospital readmissions among people living with HIV and substance use disorder. *Journal of Acquired Immune Deficiency Syndromes* **2019**, *80*, 330–341. https://doi.org/10.1097/QAI.0000000000001925.
21. Serrano, V.B.; Moore, D.J.; Morris, S.; Tang, B.; Liao, A.; Hoenigl, M.; Montoya, J.L. Efficacy of Daily Text Messaging to Support Adherence to HIV Pre-Exposure Prophylaxis (PrEP) among Stimulant-Using Men Who Have Sex with Men. *Substance Use & Misuse* **2023**, *58*, 465–469. https://doi.org/10.1080/10826084.2023.2165409.
22. Zamantakis, A.; et al. Barriers to same-day PrEP implementation. *AIDS and Behavior* **2025**. https://doi.org/10.1007/s10461-025-04898-2.
23. Paskett, E.D.; Harrop, J.P.; Wells, K.J. Patient navigation: An update on the state of the science. *CA: A Cancer Journal for Clinicians* **2011**, *61*, 237–249. https://doi.org/10.3322/caac.20111.
24. Violette, L.R.; Zewdie, K.; Gitahi, N.; Beima-Sofie, K.; Heffron, R. The pathway to delivering injectable cabotegravir for HIV prevention: A review of implementation considerations. *Implementation Science Communications* **2024**, *5*, 101. https://doi.org/10.1186/s43058-024-00637-1.
25. Randolph, S.D.; Johnson, R. A Salon-Based Intervention to Improve PrEP Uptake among Black Women. *New England Journal of Medicine* **2024**, *390*, 776–777. https://doi.org/10.1056/NEJMp2313708.
26. Seyedroudbari, S.; Ghadimi, F.; Grady, G.; et al. Structural racism and discrimination along the PrEP continuum: A systematic review. *AIDS and Behavior* **2024**, *28*, 3001–3037. https://doi.org/10.1007/s10461-024-04387-y.
27. Strathdee, S.A.; Kuo, I.; El-Bassel, N.; et al. Preventing HIV outbreaks among people who inject drugs in the United States: plus ça change, plus c'est la même chose. *AIDS* **2020**, *34*, 1997–2005. https://doi.org/10.1097/QAD.0000000000002673.
28. Deutsch, M.B.; Glidden, D.V.; Sevelius, J.; et al. HIV pre-exposure prophylaxis in transgender women: A subgroup analysis of the iPrEx trial. *The Lancet HIV* **2015**, *2*, e512–e519. https://doi.org/10.1016/S2352-3018(15)00206-4.
29. Scott, R.K.; Hull, S.J.; Kerrigan, D.; et al. Development and pilot testing of a sociostructural intervention to improve PrEP provision for Black women in the United States. *JMIR Formative Research* **2025**, p. e75922. https://doi.org/10.2196/75922.
30. Craig, P.; Dieppe, P.; Macintyre, S.; et al. Developing and evaluating complex interventions: The new Medical Research Council guidance. *BMJ* **2008**, *337*, a1655. https://doi.org/10.1136/bmj.a1655.
31. Touger, R.; Wood, B.R. A review of telehealth innovations for HIV pre-exposure prophylaxis (PrEP). *Current HIV/AIDS Reports* **2019**, *16*, 113–119. https://doi.org/10.1007/s11904-019-00430-z.
32. World Health Organization. *Task Shifting: Rational Redistribution of Tasks among Health Workforce Teams*; World Health Organization: Geneva, Switzerland, 2008.
33. Chin, M.H.; Clarke, A.R.; Nocon, R.S.; et al. A roadmap and best practices for organizations to reduce racial and ethnic disparities in health care. *Journal of General Internal Medicine* **2012**, *27*, 992–1000. https://doi.org/10.1007/s11606-012-2082-9.
