# Supplementary material for: Computational Validation of a Clinical Decision Support Algorithm for LAI-PrEP Bridge Period Navigation at UNAIDS PrEP Target Scale (21.2 Million Individuals)
Source: Viruses. 2026 Feb 13;18(2):237. doi: 10.3390/v18020237 (PMC12945109; doi:10.3390/v18020237)
Supplement: Supplementary file 1 [file viruses-18-00237-s001.zip › viruses-4063895-S3-final.pdf]

Supplementary Materials: COMPUTATIONAL VALIDATION OF A CLINICAL DECISION SUPPORT ALGORITHM FOR LAI–BRIDGE PERIOD NAVIGATION AT UNAIDS PREP TARGET SCALE (21.2 MILLION INDIVIDUALS)

Supplementary File S3

AI Readiness in Healthcare

Framework for Computational Validity and Clinical Deployment

Version 2.1 | December 2025

DOI: https://doi.org/10.5281/zenodo.17873201

Corresponding manuscript: Demidont, A.C. (2025). Validation of a Clinical Decision Support Algorithm for LAI-PrEP Bridge Period Navigation at UNAIDS PrEP Target Scale (21.2M Individuals). *Viruses*.

Purpose of This Supplement

We systematically examine whether the LAI-PrEP Bridge Period Decision Support Tool is ethically and operationally ready for clinical deployment. Rather than assuming computational validation guarantees clinical readiness, we interrogate six questions that determine whether this algorithm can be responsibly implemented: (1) Is sufficient data available? (2) Does computational precision create false confidence? (3) Can clinicians trust extrapolated parameters? (4) Does transparency enable appropriate oversight? (5) Does the system perform equitably across populations? (6) Should implementation proceed, and under what conditions? Our framework draws from best practices in responsible AI deployment, health equity, and clinical decision support systems literature.

Definition of Success: We define success simply: build an algorithm that returns interventions supporting all stakeholders during the period from LAI-PrEP prescription through first injection, to safely and equitably increase global LAI-PrEP uptake and decrease global HIV burden.

The stakeholder matrix our algorithm must satisfy is multi-tiered and often contains competing priorities (Figure S1).

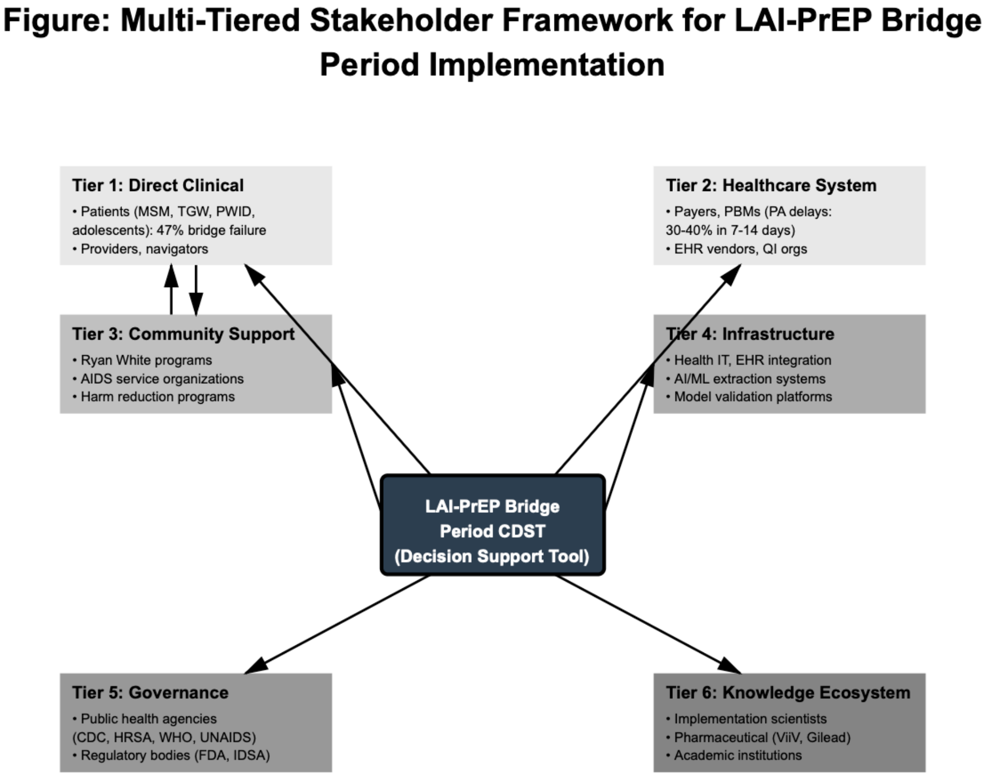

Stakeholder Tier Definitions

**Tier 1:**	Primary users in direct patient care (patients, clinicians, navigators)
**Tier 2:**	Healthcare system infrastructure (payers, quality measurement, EHR systems)
**Tier 3:**	Community organizations addressing structural barriers
**Tier 4:**	Technical platforms and AI/ML infrastructure
**Tier 5:**	Regulatory and public health governance bodies
**Tier 6:**	Research, pharmaceutical, and knowledge generation stakeholders

Figure S1. Multi-tiered stakeholder framework for LAI-PrEP bridge period clinical decision support implementation. The decision support tool operates within six interconnected stakeholder layers: (1) direct clinical care (patients, providers, navigators), (2) healthcare system infrastructure (payers, insurance authorization, quality measurement), (3) community organizations addressing structural barriers (harm reduction, Ryan White, AIDS service organizations), (4) technical infrastructure and AI/ML platforms, (5) governance and regulatory bodies (CDC, HRSA, WHO, UNAIDS, FDA), and (6) knowledge generation ecosystem (implementation scientists, academic institutions, pharmaceutical manufacturers). Bidirectional arrows indicate feedback mechanisms. Successful implementation requires alignment across all tiers, with the CDST serving as the common coordination point.

Competing Stakeholder Priorities and Algorithmic Complexity

The six-tiered stakeholder framework reveals fundamental tensions that a clinical decision support tool must simultaneously navigate. Tier 1 (direct clinical care) prioritizes individual patient outcomes and treatment adherence—clinicians need real-time recommendations that account for patients' specific barriers and lived experiences, with transparency sufficient to earn trust from populations historically harmed by medical systems. Tier 2 (healthcare systems) demands operational efficiency and cost containment—payers require evidence that interventions reduce administrative burden (particularly the 30–40% authorization delays that drive bridge period attrition) and generate favorable return-on-investment. Tier 3 (community organizations) emphasizes structural equity and community voice—harm reduction programs and AIDS service organizations require that algorithmic recommendations neither displace nor contradict community-based interventions already serving vulnerable populations. Simultaneously, Tier 4 (technical infrastructure) requires mathematical precision, reproducibility, and integration compatibility with existing EHR systems; Tier 5 (governance bodies like CDC, HRSA, UNAIDS) demands evidence suitable for policy guidelines and resource allocation decisions at population scale; and Tier 6 (knowledge ecosystem) requires that the tool advance implementation science and avoid reinforcing pharmaceutical marketing over genuine clinical need. These priorities are not merely different—they are often fundamentally opposed. Equity-focused interventions for the most vulnerable populations are frequently the most resource-intensive and administratively complex, creating tension with healthcare system efficiency goals. Individual patient-centered transparency clashes with aggregate statistical precision. Community-driven implementation sometimes contradicts standardized protocols necessary for healthcare system scalability.

The LAI-PrEP Bridge Period Decision Support Tool addresses this complexity through three architectural choices. First, explicit evidence tiering ensures that clinicians understand the confidence level underlying each recommendation: Tier 1 parameters (direct LAI-PrEP data) receive higher priority in clinical decision-making than Tier 3 parameters (cross-field extrapolation), while making this distinction transparent to all stakeholders. Second, the mechanism diversity scoring algorithm prevents the tool from simply recommending whichever single intervention has the largest effect size; instead, it mandates multi-component interventions addressing complementary barriers, reflecting the reality that vulnerable populations require diverse supports rather than single-strategy solutions. Third, the configuration-driven architecture externalizes all clinical parameters, enabling different healthcare systems, community organizations, and regulatory bodies to adapt the tool to local context without requiring code modifications. This configurability acknowledges that a one-size-fits-all algorithm cannot simultaneously satisfy Tier 1 through Tier 6 priorities. The tool succeeds not by resolving these tensions, but by making them explicit and providing the scaffolding within which different stakeholders can negotiate appropriate local solutions.

1. Question 1: Data Availability

Is sufficient high-quality data available to build, validate, and continuously monitor the model?

Response: Yes and No. Data gaps exist despite voluminous structured epidemiology data.

Computational algorithms require data to learn from, validate against, and monitor for performance degradation. This foundational question addresses whether the data ecosystem supports responsible AI deployment.

https://doi.org/10.3390/v18020237

Current Best Practices vs. Real-World Implementation Gap

Sexual health history taking has established best practices: the CDC recommends systematic structured documentation using the 5Ps framework (Partners, Practices, Past STDs, Protection, Pregnancy) at initial and annual prevention visits [1]. We argue a 6th P (Power dynamics) should be included in routine sexual history taking. Sexual histories are vital signs for HIV prevention. The National LGBTQIA+ Health Education Center specifies that sexual health templates be embedded in electronic medical records to ensure standardization and systematic capture [2]. EMR documentation standards require accuracy and clinical objectivity while respecting patient preferences about what information is recorded [3]. Yet, structured sexual health data collection remains uncommon in practice. Unlike chronic disease management (where diabetes templates systematically capture A1C, blood pressure, medication adherence), sexual health templates are absent from most "out of the box" EHRs. System priorities vary significantly between large academic medical centers, community health centers, family medicine practices, and urgent care. For young, healthy PrEP users with minimal baseline healthcare contact, EHRs have no established infrastructure for sexual health tracking. The result: longitudinal sexual history records, even in family planning settings, are unstructured or missing.

Data Exist But Are Inaccessible

Structured epidemiology databases tracking HIV, STI, and other reportable infections exist at local, state, and federal levels. The CDC's National Notifiable Diseases Surveillance System (NNDSS), state health department disease registries, and local public health agencies maintain extensive structured data on HIV diagnoses, STI diagnoses, and prevention services. These databases represent decades of standardized, longitudinal surveillance infrastructure.

However, these data are *systematically siloed and largely inaccessible outside their originating agencies*. Access is restricted by:

- **Legal protections:** Enhanced privacy laws for HIV/STI data prevent data sharing across institutional boundaries
- **Technical barriers:** Legacy systems with incompatible formats, no standardized APIs
- **Governance silos:** Data owned by agencies that prioritize surveillance over research
- **IRB/regulatory complexity:** Data use agreements requiring months of negotiation

The result: While the epidemiologic infrastructure for tracking HIV exists, bridge period outcome data cannot be accessed by clinicians, program planners, or researchers attempting to improve LAI-PrEP implementation.

Evidence Synthesis Process

We synthesized evidence from n>15,000 participants across four major LAI-PrEP trials (HPTN 083, HPTN 084, PURPOSE-1, PURPOSE-2) [4] to establish population-specific baseline success rates. This direct LAI-PrEP data (Tier 1 evidence) forms the core of our model parameters. However, we also relied on evidence extrapolation from oral PrEP cascade studies (Tier 2) and cross-field implementation literature (Tier 3).

A 2024 systematic review by Kamitani et al., conducted by CDC's Prevention Research Synthesis Project, evaluated the entire published PrEP intervention literature against established Best Practices criteria [4]. The findings are stark:

- **3,974** PrEP-related citations in CDC's cumulative database
- **266** full-text articles screened for eligibility
- **24** studies met basic eligibility criteria (0.6%)
- **9** met Best Practices standards (0.2% of initial citations)

https://doi.org/10.3390/v18020237

Of 3,974 funded PrEP studies, 99.8% failed to meet CDC's own quality criteria.

The PWID Evidence Vacuum

Of 24 published studies meeting basic PrEP intervention criteria, zero were conducted with people who inject drugs. Zero. The population with highest HIV burden and lowest treatment access has zero evidence-based interventions in the published literature. An algorithm trained on this literature will inherit this erasure—not through malice, but through the mathematical impossibility of learning patterns that don't exist in the training data. This is not a parameter we can extrapolate. This is a population we must validate separately, with community partnership from the outset.

Implications for Deployment

Until prospective data collection infrastructure is established and pilot validation is complete, the tool could be used for decision-support in research settings with systematic outcome tracking, training and clinician education, and resource planning and policy development.

The tool is not ready for unrestricted deployment without real-world prospective validation (target: $n \geq 500$ patients, ≥ 10 sites), demonstrated performance meeting or exceeding model predictions, infrastructure to capture bridge period outcomes, and equity-stratified outcome monitoring.

2. Question 2: External Validity

Does computational precision create false confidence?

Response: Yes, without algorithmic transparency.

High computational precision (our model achieves ±0.018 pp at 21.2M scale) can create false confidence in external validity—the assumption that predictions will match real-world outcomes.

Computational vs. Clinical Validity

We demonstrated computational precision through progressive validation across four scales (1K, 1M, 10M, 21.2M), with 144-fold precision gain and 100% test pass rate across 18 edge cases. However, we explicitly distinguished:

- **Computational validity:** The algorithm produces stable, mathematically sound predictions given fixed input parameters
- **Clinical validity:** Algorithm predictions match actual patient outcomes in diverse real-world settings

Even with high computational precision, three sources of uncertainty affect real-world accuracy: (1) Parameter estimation uncertainty from diverse evidence tiers, (2) Implementation fidelity variation across clinical settings, (3) Context-specific effect modification not captured in the model.

Bounding Uncertainty Through Prospective Validation

We propose a calibration framework where observed improvements of 50–100% of model predictions indicate successful validation. Even if real-world effects are 50% of modeled predictions, this would represent 2.1 million additional successful transitions globally—a substantial public health impact. Observed effects <50% of predictions would trigger systematic investigation, parameter recalibration, and model refinement.

https://doi.org/10.3390/v18020237

Implications for Deployment

Computational validation establishes algorithmic readiness for testing, not clinical readiness for unrestricted deployment. Prospective multi-site validation is essential before widespread implementation.

3. Question 3: Explainability

Can clinicians trust the extrapolated parameters?

Response: Yes, when provided explainable algorithms with potential for meaningful clinical impact.

Parameters embedded in clinical decision support must be transparent and trustworthy. When parameters derive from extrapolation rather than direct evidence, clinicians need to understand the evidence tiers and make informed judgments about applicability.

Evidence Tier Classification

We categorized all 21 intervention parameters by evidence source:

Table S1. Evidence Foundation Summary: LAI-PrEP Bridge Tool Parameters by Evidence Tier

Tier	Source	Interventions (n)	Confidence
**Tier 1:** Direct LAI-PrEP	HPTN 083, HPTN 084, PURPOSE	8	Highest
**Tier 2:** HIV prevention analog	Oral PrEP, HIV care engagement	9	Moderate
**Tier 3:** Cross-field extrapolation	Cancer care, chronic disease	4	Requires validation
**Total**		21	

Why Extrapolation Matters

Tier 2 and Tier 3 parameters are necessary because not all populations have LAI-PrEP-specific implementation data, not all intervention strategies have been tested in LAI-PrEP contexts, and waiting for perfect evidence would mean never deploying the tool. However, extrapolation carries uncertainty that must be managed transparently.

Transparency as Trust-Building

The tool's external configuration documents every parameter with evidence tier classification, source citations, effect size ranges, and version history. This transparency allows clinicians and program planners to understand the evidentiary foundation, make informed judgments about local applicability, modify parameters if local data suggest different effect sizes, and adapt the tool as new LAI-PrEP implementation data emerge.

Implications for Deployment

Before clinical deployment,we recommend review the evidence tier classification, consider whether Tier 2/Tier 3 parameters apply in their local context, collect local outcome data to validate or refine parameters, and share findings to improve the evidence base for all users.

4. Question 4: Interpretability

Does transparency enable appropriate clinical oversight?

Response: Yes. However, transparency alone does not prevent misuse.

Transparent algorithms enable clinician understanding and appropriate override. However, research reveals a problem: *automation bias*—the tendency of users to over-rely

on automated systems and accept their output without sufficient scrutiny [5]—is well-documented across healthcare contexts.

Transparent Architecture

The LAI-PrEP Bridge Tool uses an explicitly interpretable structure with four stages: (1) Barrier Identification, (2) Population Risk Stratification, (3) Mechanism Diversity Scoring, (4) Combined Effect Calculation. At each stage, clinicians can see the reasoning, question assumptions, override recommendations, and understand why specific suggestions were made.

The Automation Bias Problem

Automation bias manifests as two types of errors: omission errors (failing to notice algorithmic errors) [6] and commission errors (inappropriately trusting algorithm recommendations despite conflicting clinical evidence). Healthcare environments exacerbate these risks: clinicians face fatigue, understaffing, high workload, and time constraints that incentivize reliance on automation over cognitive effort [7]. Conversely, "alert fatigue" can lead to dismissal bias when clinicians begin to distrust systems due to high false-positive rates.

Implementation should Include:

- **Clinician Training on Algorithm Limitations:** Explicit exposure to automation failures reduces complacency and misuse [6]
- **Organizational Policies Supporting Clinical Override:** Low-friction override mechanisms support appropriate clinical judgment
- **Regular Audits of Algorithm Use:** Detect misuse patterns through systematic monitoring by demographic subgroup
- **Continuous Feedback to Algorithm Developers:** Close feedback loops detect concept drift and real-world performance degradation [8]

Implications for Deployment

Before clinical deployment: review the transparent algorithm architecture, establish override procedures with documentation requirements, implement targeted clinician training on domain-specific limitations, and build feedback mechanisms tracking algorithm use, override rates, outcomes, and patterns of potential misuse.

5. O'Neil's Framework: Why Algorithm Bias is Structural, Not Incidental

Cathy O'Neil describes "weapons of math destruction" [9] (WMDs) as algorithms that are opaque, unregulated, and difficult to contest while being scaled to affect large populations. They do not eliminate bias; they embed existing societal biases into code, creating the false appearance of mathematical objectivity while masking discrimination.

A mechanism O'Neil identifies is the *feedback loop*: as algorithms influence human decisions and generate data that feed back into the system, they reinforce their own biases [9]. An LAI-PrEP algorithm trained on data from oral PrEP clinical trials, published implementation studies, and insurance databases would quickly learn to generate highly accurate success predictions for White MSM in Western settings while producing wildly inaccurate predictions for racial and gender minorities, solely based on historical exclusion and access barriers.

This bias would undermine the implementation barrier LAI-PrEP solves for the populations LAI-PrEP could benefit most.

https://doi.org/10.3390/v18020237

6. Question 5: Equity

Does the system perform equitably across populations, or do population averages mask disparities?

Response: Population-level analysis masks substantial within-group disparities. Equity-focused implementation with stratified monitoring is non-negotiable.

Three Equity Assessment Dimensions

1. Differential Calibration: Does the algorithm's accuracy vary by demographic group? The model's population-specific validation shows comparable accuracy across populations. However, real-world calibration should be measured prospectively: Does the algorithm predict 25% success rate equally accurately for Black women as for White MSM? Differential calibration errors could systematically misdirect resources.

2. Differential Benefit: Do interventions work equally well across populations? Our modeling suggests PWID benefit most from interventions (265% improvement). This could indicate either that interventions are particularly effective for populations with lowest baseline success, OR that extrapolated effect sizes for PWID interventions are inflated. Prospective validation must measure intervention effectiveness separately by population.

3. Outcome Disparities: Even if algorithm and interventions perform equally, do outcomes remain disparate due to structural factors? If the algorithm recommends transportation assistance but transportation programs only exist in urban centers, rural patients cannot benefit.

Implementation Safeguards

To ensure equity-focused implementation:

- **Stratified Outcome Monitoring:** Track bridge period success separately by race, ethnicity, gender identity, socioeconomic status, and geography with automated alerts if disparities widen
- **Intervention Accessibility Audits:** Verify that recommended interventions are actually available and accessible to all populations
- **Equitable Resource Allocation:** Populations with lowest baseline success rates should receive proportionally more resources
- **Community Engagement:** Include community representatives from key populations in algorithm governance, validation design, and implementation
- **Continuous Feedback:** Regular review of outcomes by population subgroup with explicit discussion of why disparities exist

Implications for Deployment

We suggests health systems commit to stratified outcome monitoring by demographic groups if deploying this algorithm, create infrastructure ensuring recommended interventions are equitably accessible, organize community advisory boards representing key populations, and regular (quarterly minimum) run equity audits with review regularly with circuit-breaker procedures if disparities worsen.

7. Question 6: Readiness

Should implementation proceed, and under what conditions?

Response: Suitable for pilot clinical validation with clear stopping rules.

Computational validity does not equal clinical readiness. Even algorithmically sound systems require careful staged implementation with clear stopping rules, infrastructure development, and continuous monitoring.

https://doi.org/10.3390/v18020237

Readiness Assessment

Table S2. Readiness Assessment: Current Status

Readiness Domain	Status	Required Before Deployment?
Computational validation	Complete	No
Evidence synthesis	Complete	No
Interpretability	Demonstrated	No
Equity framework	Designed	Yes
Data infrastructure	Absent	Yes
Prospective validation	Not started	Yes
Implementation protocols	Designed	Yes
Clinician training	Not developed	Yes
Governance structure	Not established	Yes

Status Key: "No" indicates the domain is sufficient for pilot testing but may need refinement. "Yes" indicates the domain is for broader deployment and must be established during pilot phase.

Recommended Staged Implementation Pathway

Stage 1: Pilot Validation (6–12 months)

Sites: 2–3 early adopters with research capacity and existing bridge period tracking. Participants: 50–100 patients. Goals: Proof-of-concept, identify implementation barriers, refine data collection procedures. Success criteria: >80% outcome capture, no safety signals, clinician satisfaction >70%.

Stage 2: Multi-Site Validation (12–24 months)

Sites: 10–15 community health centers, specialty sexual health clinics. Participants: 500–1,000 patients. Goals: Prospective validation with population-stratified subgroup analyses, infrastructure refinement. Success criteria: Real-world effectiveness 50–100% of modeled predictions, equitable outcomes, sustained clinician engagement.

Stage 3: Scaled Implementation (24+ months)

Sites: Interested healthcare systems with demonstrated data collection capacity. Participants: Open-ended enrollment. Goals: Real-world effectiveness monitoring, parameter refinement, continuous improvement. Success criteria: Sustained effectiveness, maintained equity, clinician adoption >50%.

Stopping Rules and Circuit Breakers

Implementation should be paused or modified if:

- **Safety Signal:** Unexpected adverse outcomes (HIV acquisition, unintended consequences)
- **Efficacy Failure:** Real-world effectiveness <20% of modeled predictions
- **Equity Failure:** Outcome disparities widen or algorithm performs differentially by demographic group
- **Clinician Rejection:** Adoption rate <30% after 6 months in stage with intensive training
- **Infrastructure Failure:** Unable to collect >70% of required outcome data

When stopping rules are triggered, pause implementation, investigate, and adjust before proceeding.

Implications for Deployment

The tool is ready for decision-support research in clinical settings with systematic outcome tracking, clinician training and education about bridge period barriers, and resource planning and policy discussion.

https://doi.org/10.3390/v18020237

The tool is *not* ready for unrestricted deployment in routine clinical practice, autonomous decision-making without human oversight, use in settings without capacity for outcome monitoring, or deployment without community engagement and equity monitoring.

Synthesis: The AI Readiness Paradox

This supplement has systematically addressed six questions, revealing a central paradox: The more rigorously we validate computational soundness, the more we expose clinical uncertainty. We have achieved mathematical precision (±0.018 pp at 21.2M scale) while acknowledging fundamental gaps in evidence quality, real-world calibration, and health equity. This is not a weakness to hide. It is the foundation of responsible deployment.

The tool is computationally ready (✓) but clinically unproven (×). It is theoretically sound (✓) but missing accessible data infrastructure (×). It is ethically designed (✓) but cannot guarantee equitable outcomes without continuous monitoring (×).

These tensions cannot be resolved in advance. They must be managed through staged implementation, clear stopping rules, and genuine community partnership. An algorithm that appears to have all answers is dangerous. One that names its limitations and invites scrutiny is trustworthy.

Answering all six questions honestly—acknowledging limitations and gaps, not just successes—is the foundation of responsible AI deployment in healthcare.

Reference: A.C Demidont, DO(2025). Computational Validation of a Clinical Decision Support Algorithm for Long-Acting Injectable PrEP Bridge Period Navigation at UNAIDS Global Target Scale. *Viruses*

1. Centers for Disease Control and Prevention. A Guide to Taking a Sexual History, 2021.
2. Bogaert, E.; Roels, R. Sexual health in patient care: Shortcomings in medical training and experienced barriers in sexual history taking. *BMC Medical Education* **2025**, *25*, 338. https://doi.org/10.1186/s12909-025-06850-3.
3. American Health Information Management Association. Definition of the Health Record for Legal Purposes. *Journal of AHIMA* **2001**, *72*, 88.
4. Kamitani, E.; Higa, D.H.; Crepaz, N.; et al. Identifying best practices for increasing linkage to, retention in, and reengagement with HIV medical care: A systematic review. *AIDS and Behavior* **2024**, *28*, 2340–2349. https://doi.org/10.1007/s10461-024-04332-z.
5. Parasuraman, R.; Riley, V. Humans and automation: Use, misuse, disuse, abuse. *Human Factors* **1997**, *39*, 230–253. https://doi.org/10.1518/001872097778543886.
6. Bahner, J.E.; Hüper, A.D.; Manzey, D. Misuse of automated decision aids: Complacency, automation bias and the impact of training experience. *International Journal of Human-Computer Studies* **2008**, *66*, 688–699. https://doi.org/10.1016/j.ijhcs.2008.06.001.
7. Sittig, D.F.; Wright, A.; Osheroff, J.A.; Middleton, B.; Teich, J.M.; Ash, J.S.; Bates, D.W. Grand challenges in clinical decision support. *Journal of Biomedical Informatics* **2008**, *41*, 387–392. https://doi.org/10.1016/j.jbi.2007.09.003.
8. Agarwal, R.; Bjarnadottir, M.V.; Rhue, L.; Dugas, M.; Crowley, K.; Clark, J.; Gao, G. Addressing algorithmic bias and the perpetuation of health inequities: An AI bias-aware framework. *Health Policy and Technology* **2023**, *12*, 100702. https://doi.org/10.1016/j.hlpt.2022.100702.
9. O'Neil, C. *Weapons of Math Destruction: How Big Data Increases Inequality and Threatens Democracy*; Crown Publishing Group: New York, NY, USA, 2016.
