# Supplementary material for: Computational Validation of a Clinical Decision Support Algorithm for LAI-PrEP Bridge Period Navigation at UNAIDS PrEP Target Scale (21.2 Million Individuals)
Source: Viruses. 2026 Feb 13;18(2):237. doi: 10.3390/v18020237 (PMC12945109; doi:10.3390/v18020237)
Supplement: Supplementary file 1 [file viruses-18-00237-s001.zip › viruses-4063895-S5-final.pdf]

Supplementary Materials: COMPUTATIONAL VALIDATION OF A CLINICAL DECISION SUPPORT ALGORITHM FOR LAI–BRIDGE PERIOD NAVIGATION AT UNAIDS PREP TARGET SCALE (21.2 MILLION INDIVIDUALS)

Supplementary File S5

Additional Tables for Computational Validation

Population-Specific Baselines, Regional Analysis, Risk Stratification, and Barrier Impact

Version 2.1 | December 2025 | Corresponds to validation at UNAIDS global scale (n=21.2M)

Corresponding manuscript: Demidont, A.C. (2025). Validation of a Clinical Decision Support Algorithm for LAI-PrEP Bridge Period Navigation at UNAIDS PrEP Target Scale (21.2M Individuals). *Viruses*.

Table S1: Population-Specific Baseline Success Rates at UNAIDS Global Scale (n=21.2M)

Table S1. Population-specific bridge period completion success rates (baseline without interventions) at UNAIDS 2025 global target scale. Data represents 21.2 million synthetic patients stratified by population category. Baseline rates represent predicted success probability for receiving first LAI-PrEP injection during bridge period without evidence-based interventions. Published ranges derived from clinical trial outcomes (HPTN 083, HPTN 084, PURPOSE-1/2) and real-world implementation studies.

Population Category	n (21.2M) Scale	Predicted Rate	95% CI	SE Range	Published	Source
Men who have sex with men (MSM)	2.97M (14%)	33.11%	33.07–33.15%	0.02%	35–40%	HPTN 083 trial
General population	4.24M (20%)	31.22%	31.18–31.26%	0.02%	30–35%	CAN implementation
Transgender women	1.27M (6%)	28.46%	28.42–28.50%	0.02%	30–35%	HPTN 083 sub-analysis
Cisgender women	5.30M (25%)	24.10%	24.07–24.13%	0.015%	25–30%	HPTN 084, PURPOSE-1
Pregnant/lactating	1.91M (9%)	24.11%	24.08–24.14%	0.015%	25–30%	Clinical extrapolation
Adolescents (16–24y)	2.65M (13%)	16.34%	16.31–16.37%	0.015%	15–25%	Adolescent PrEP cascade
People who inject drugs (PWID)	2.88M (13%)	10.36%	10.33–10.39%	0.015%	10–20%	Harm reduction literature
**Overall Global Average**	**21.2M**	**23.96%**	**23.94–23.98%**	**0.009%**	–	–

Note: SE = standard error; CI = confidence interval. Population distribution reflects UNAIDS 2025 regional prevalence estimates. MSM baseline includes transgender women (HPTN 083 enrollees). Adolescent rates extrapolated from oral PrEP cascade literature. PWID rates based on harm reduction integration literature given low direct LAI-PrEP

implementation data in this population. See main manuscript Methods section for complete evidence source mapping.

*** References:** HPTN 083 [1]; HPTN 084 [2]; PURPOSE-1 [3]; PURPOSE-2 [4]; CAN implementation [5]; Adolescent PrEP cascade [6]; Harm reduction literature [7,8]; UNAIDS estimates [9].

Table S2: Regional Analysis at UNAIDS Global Scale—Baseline and Intervention Effects

Table S2. Regional variation in bridge period success rates and intervention effectiveness at UNAIDS 2025 global target scale. Sub-Saharan Africa (SSA) serves 62% of global PrEP-eligible population but demonstrates lowest baseline success. Evidence-based interventions show greatest absolute and relative improvement in regions with lowest baseline, demonstrating equity-focused benefits.

Region	n (21.2M)	% of Global	Baseline Success	95% CI	With Interventions	Absolute Improvement	Relative Improvement
Sub-Saharan Africa	13.14M	62%	21.69%	21.67–21.71%	41.51%	+19.82 pp	+91.4%
North America	3.82M	18%	29.33%	29.30–29.36%	45.18%	+15.85 pp	+54.0%
Latin America/-Caribbean	2.04M	9%	25.44%	25.41–25.47%	43.72%	+18.28 pp	+71.8%
Europe/Central Asia	1.27M	6%	29.33%	29.29–29.37%	45.33%	+16.00 pp	+54.6%
Asia/Pacific	1.06M	5%	24.12%	24.08–24.16%	43.18%	+19.06 pp	+79.0%
**Global Average**	**21.2M**	**100%**	**23.96%**	**23.94–23.98%**	**43.50%**	**+19.54 pp**	**+81.6%**

Note: Regional stratification reflects current global PrEP epidemiology and UNAIDS 2025 scale-up targets. SSA equity gap: 7.64 pp (SSA 21.69% vs. Europe/Central Asia 29.33%). Despite lowest baseline, SSA shows greatest absolute improvement (+19.82 pp) and strong relative improvement (+91.4%), demonstrating that targeted interventions can reduce rather than widen health equity gaps. pp = percentage points; CI = confidence interval.

***References:** UNAIDS Global AIDS Update [9]; Health equity framework [10].

Table S3: Structural Barrier Impact Analysis—Dose-Response Relationship at UNAIDS Global Scale

Table S3. Structural barrier dose-response relationship: bridge period success rate declines linearly with increasing number of co-occurring barriers. At 21.2M scale, 85.7% of patients faced at least one barrier; 43.1% faced 3+ barriers with <15% predicted success without interventions. Barriers modeled using multiplicative combination method reflecting synergistic effects.

Number of Barriers	Patients (21.2M)	% of Population	Success Rate (baseline)	95% CI	Decrease per Barrier (pp)
0 barriers	3.05M	14.4%	44.02%	43.98–44.06%	Baseline
1 barrier	5.53M	26.1%	36.19%	36.15–36.23%	−7.83 pp
2 barriers	4.99M	23.5%	28.52%	28.48–28.56%	−7.67 pp
3 barriers	3.67M	17.3%	21.82%	21.78–21.86%	−6.70 pp
4 barriers	2.24M	10.6%	16.35%	16.31–16.39%	−5.47 pp
5+ barriers	1.82M	8.6%	12.14%	12.10–12.18%	−4.21 pp
**At least 1 barrier**	**18.25M**	**85.6%**	**24.37%**	**24.35–24.39%**	**Average: −7.74 pp**
**3+ barriers**	**9.15M**	**43.1%**	**16.77%**	**16.74–16.80%**	**Clinical concern**

Note: Linear regression of barrier count versus success rate: R^2=0.998, slope=−7.74 pp per barrier. Multiplicative model reflects synergistic barrier effects: each additional barrier proportionally reduces remaining success probability rather than adding linearly. “Clinical concern” threshold set at <15% success rate, achieved at 3+ barriers. Barriers include: transportation, insurance delays, medical mistrust, stigma, childcare, appointment scheduling, confidentiality concerns, testing delays, provider availability, pharmacy access, language barriers, housing instability, and food insecurity. pp = percentage points.

*__References:__ Transportation barriers [11]; Structural racism and PrEP [12]; Stigma in HIV interventions [13]; PrEP implementation barriers [14,15].

Table S4: Risk Stratification Distribution at UNAIDS Global Scale

Table S4. Risk stratification distribution reflecting predicted attrition risk based on baseline success rate and barrier count. Four-category risk model: Very Low Risk (>40% success), Low Risk (30–40%), High Risk (15–30%), Very High Risk (<15%). Distribution at 21.2M scale shows 65.3% classified as Very High Risk, reflecting concentration of barriers in vulnerable populations and Sub-Saharan Africa (62% of sample).

Risk Category	Success Rate Range	Patients (21.2M)	% of Population	Clinical Interpretation	Priority
Very Low Risk	>40%	0.82M	3.9%	Minimal attrition expected; standard care sufficient	Monitoring only
Low Risk	30–40%	2.45M	11.6%	Moderate attrition risk; basic navigation recommended	Routine support
High Risk	15–30%	4.42M	20.9%	Substantial attrition risk; multi-component interventions strongly recommended	Targeted intervention bundle
Very High Risk	<15%	13.85M	65.3%	Critical attrition risk; comprehensive intervention bundle essential	Intensive multi-modal support
**Total**	**–**	**21.2M**	**100%**	–	–

Note: Risk stratification based on population category, barrier count, and regional context. Very High Risk concentration (65.3%) reflects: (1) Sub-Saharan Africa 62% representation with lower baseline success (21.69%); (2) PWID population 13% with lowest baseline success (10.36%); (3) Adolescents 13% with 16.34% baseline. Risk categories map to recommended intervention intensity: Very Low Risk=monitoring; Low Risk=routine navigation; High Risk=targeted bundles; Very High Risk=intensive multi-modal support. Clinical significance: the 65.3% Very High Risk classification indicates that evidence-based bridge period interventions are essential for majority of global PrEP-eligible population.

***References:** Patient navigation outcomes [16]; Panel management and PrEP initiation [17]; Risk stratification frameworks [18].

Table S5: Individual Barrier Impact Weights and Clinical Implementation Thresholds

Table S5. Individual structural barrier impact on bridge period success: estimated percentage point reduction in success probability for each barrier type. Impact weights derived from published implementation literature, patient navigation studies, and clinical expert consultation. Used in multiplicative combination model where each barrier proportionally reduces remaining success probability.

Barrier Type	Impact Weight (pp reduction)	Evidence Tier	Population Prevalence	Implementation Level
Transportation/Logistics	−15%	2	25%	Moderate
Insurance Authorization Delays	−12%	2	40%	Moderate
Medical Mistrust/Stigma	−10%	2	30–50%	Medium
Appointment Scheduling Conflicts	−8%	3	35%	Low
Confidentiality/Privacy Concerns	−8%	3	20–40%	Low
Childcare/Dependent Care	−7%	2	15–30%	Medium
Testing Delays/Capacity	−6%	2	20%	Low
Provider Availability	−5%	3	25%	Low
Pharmacy Access	−5%	3	15%	Low
Language Barriers	−4%	3	10–15%	Low
Housing Instability	−10%	2	8–12%	High
Food Insecurity	−6%	3	5–10%	High
Discrimination/Persecution Risk	−12%	2	15–35%	High
**Maximum Observable (13 barriers combined)**	−98%	–	–	–

Note: Impact weights represent individual barrier effects assuming multiplicative combination. Evidence Tiers: Tier 1=Direct LAI-PrEP data; Tier 2=HIV prevention or healthcare analog; Tier 3=Cross-field extrapolation. Population prevalence estimates derived from patient navigation studies, HPTN trial enrollment data, and implementation literature. Impact weights clinically validated against published cascade data (Tier 1) and conservatively estimated for barriers lacking direct LAI-PrEP evidence. Implementation level reflects resource requirements for barrier mitigation (Low=<$100/patient; Moderate=$100–300/patient; High=>$300/patient). Multiplicative model prevents mathematical impossibilities (success probability never reaches exactly zero even with all barriers present; maximum combined reduction limited to 98%).

***References:** Implementation literature [14,15]; Patient navigation studies [16,19]; HPTN trial data [1,2]; Barrier prevalence [20,21].

Table S6: Convergence Validation—Success Rate Stability Across Progressive Scales

Table S6. Success rate convergence across progressive validation scales (1K, 1M, 10M, 21.2M), demonstrating algorithmic stability and improved precision. Mean success rates stabilized by 1M scale (27.7%). Apparent shift to 23.96% at 21.2M reflects regional stratification (62% Sub-Saharan Africa) rather than algorithmic instability.

Population	Tier 1 (1K)	Tier 2 (1M)	Tier 3 (10M)	Tier 4 (21.2M)	Consistency Ratio (T4/T2)
MSM	30.4%	35.7%	37.6%	33.11%	0.93
General population	28.1%	35.7%	35.7%	31.22%	0.88
Transgender women	26.6%	32.8%	32.8%	28.46%	0.87
Cisgender women	19.6%	28.1%	28.1%	24.10%	0.86
Pregnant/lactating	22.1%	28.0%	28.1%	24.11%	0.86
Adolescents	15.5%	19.4%	19.4%	16.34%	0.84
PWID	9.5%	12.2%	12.1%	10.36%	0.85
**Global Average**	**21.7%**	**27.7%**	**27.7%**	**23.96%**	**0.87**

Note: Consistency ratio = Tier 4 (21.2M) / Tier 2 (1M). Ratios 0.84–0.93 demonstrate stable relative relationships across scales. Absolute differences reflect: (1) Tier 1 (1K) sampling variability (SE=$\pm$2.6 pp); (2) Tier 2–3 uniform North American/European distribution; (3) Tier 4 regional stratification with 62% Sub-Saharan Africa. Population-specific ranking remains constant across all tiers (MSM highest, PWID lowest), confirming algorithmic stability despite regional composition changes.

https://doi.org/10.3390/v18020237

Table S7: Intervention Effectiveness Across Population Groups—Absolute and Relative Improvements

Table S7. Intervention bundle effectiveness stratified by population category, showing both absolute improvement (percentage points) and relative improvement (percent change from baseline). Greatest absolute improvements in populations with highest barriers (PWID, adolescents); interventions demonstrably reduce rather than widen health equity gaps. All interventions applied as optimized diverse bundles using mechanism diversity scoring algorithm.

Population	Baseline Success	With Interventions	Absolute Improvement	Relative Improvement	Number of Interventions	Target Barriers
PWID	10.36%	37.82%	+27.46 pp	+265%	6	Transportation, mistrust, SSP
Adolescents	16.34%	40.30%	+23.96 pp	+147%	5	Confidentiality, navigation, transport
Cisgender women	24.10%	48.06%	+23.96 pp	+99%	5	Transport, childcare, navigation
Pregnant/lactating	24.11%	39.44%	+15.33 pp	+64%	4	Antenatal integration, navigation
Transgender women	28.46%	43.82%	+15.36 pp	+54%	4	Anti-discrimination, peer support
General population	31.22%	46.57%	+15.35 pp	+49%	3	Navigation, testing, insurance
MSM	33.11%	48.46%	+15.35 pp	+46%	3	Same-day switching, testing, navigation
**Global Average**	**23.96%**	**43.50%**	**+19.54 pp**	**+81.6%**	**5**	**Multi-modal bundles**

Note: Intervention bundles generated using mechanism diversity scoring algorithm to optimize complementary mechanisms while preventing redundancy. PWID bundle (6 interventions): patient navigation, peer navigation, transportation support, medical mistrust intervention, harm reduction integration, mobile delivery. Bundle diversity reflects: multiple access barriers, highest intervention response, and comprehensive mechanism coverage. SSP = syringe service program integration. Global average uses weighted mean across 21.2M patient distribution. Greatest relative improvements occur in populations with lowest baseline success, directly addressing equity gaps.

*[*]**References:** Health equity framework [10]; PWID harm reduction [22]; Cisgender women interventions [20,23]; Transgender women PrEP [24]; Patient navigation [16,25].

Table S8: Healthcare Setting Variation in Success Rates

Table S8. Bridge period success rate variation by healthcare delivery setting type at UNAIDS global scale. Setting-specific variation reflects infrastructure availability, navigator capacity, structural support resources, and integration with other services (e.g., harm reduction for PWID, reproductive health for women). Specialty HIV clinics and community health centers show highest baseline success; mobile and syringe service programs show lowest baseline but greatest intervention responsiveness.

Healthcare Setting Type	Patients (21.2M)	Baseline Success	With Interventions	Relative Improvement	Infrastructure Level
Specialty HIV clinics	3.18M (15%)	36.5%	50.2%	+37.5%	High
Hospital-based ID services	2.54M (12%)	34.2%	48.8%	+42.7%	High
Community health centers	4.24M (20%)	29.4%	45.3%	+54.1%	Medium
Sexual health clinics	2.65M (13%)	27.8%	44.1%	+58.6%	Medium
Family medicine practices	3.29M (15%)	24.3%	41.8%	+72.0%	Low-Medium
Mobile health units	2.54M (12%)	15.2%	38.4%	+152.6%	Low
Harm reduction/SSP	2.04M (10%)	10.8%	36.2%	+235.2%	Low
Pharmacy-based	0.85M (4%)	22.1%	39.5%	+78.7%	Low
**Overall Average**	**21.2M**	**23.96%**	**43.50%**	**+81.6%**	–

Note: Setting distribution reflects current Ryan White HIV/AIDS Program (US) and WHO differentiated service delivery models (international). Relative improvement inversely correlated with baseline success: low-baseline settings (mobile, harm reduction) show greatest relative improvement, reflecting substantial unmet need and intervention responsiveness. Mobile and harm reduction settings disproportionately serve PWID and adolescents with highest barriers. ID = infectious disease; SSP = syringe service program. Infrastructure level reflects: navigator availability, structural support resources, integration with multi-services, and established PrEP experience.

***References:** Ryan White Program implementation [26]; WHO task shifting and service delivery [27]; Differentiated PrEP delivery [28,29]; Telehealth PrEP [30].

Table S9: Comprehensive Edge Case Testing Results

The LAI-PrEP Bridge Period Decision Support Tool was validated against 18 edge cases spanning clinical extremes, mathematical validity, mechanism diversity, data export, and error handling. All tests passed (100% pass rate), demonstrating algorithmic robustness across the full clinical spectrum.

S9.1 Edge Case Specifications and Results

Table S9. Edge Case Testing: Complete Specifications and Results (n=18 tests). All tests executed using pytest framework with automated validation of expected behaviors.

#	Category	Test Name	Description	Expected Behavior	Result
**Clinical Edge Cases (n=9)**					
1	Clinical Extreme	Maximum Barriers	PWID patient with 7 barriers (housing, transport, substance use, legal, mistrust, ID, discrimination)	Valid assessment; very high risk classification; PWID-specific interventions recommended	Pass

Continued on next page

Table S9 – *Continued from previous page*

#	Category	Test Name	Description	Expected Behavior	Result
2	Clinical Extreme	Conflicting Signals	MSM on oral PrEP but no recent HIV test	Prioritize oral-to-injectable transition AND accelerated testing; maintain $>$50% success rate	Pass
3	Clinical Extreme	Adolescent Privacy	17-year-old with parental insurance, privacy concerns, transportation barriers	High/very high risk; recommend navigation and transportation support; include adolescent-specific guidance	Pass
4	Clinical Extreme	Best Case (Zero Barriers)	MSM on oral PrEP, recent test, LGBTQ center, insured, has transportation	$\geq$85% success rate; low risk; recommend same-day switching as critical priority; $\leq$3 day bridge duration	Pass
5	Clinical Extreme	Discontinued Re-engagement	Cisgender woman who discontinued oral PrEP, has childcare/mistrust barriers	Reference discontinued status; recommend childcare support and navigation	Pass
6	Clinical Extreme	Pregnant Individual	Pregnant patient with childcare, transportation, competing priorities	$<$60% success rate; recommend barrier-specific interventions	Pass
7	Clinical Extreme	Uninsured Patient	Uninsured PWID with housing instability	Very high risk; recommend coverage assistance and SSP integration	Pass
8	Clinical Extreme	Extreme Age (Young)	16-year-old adolescent	Valid assessment within probability bounds	Pass
9	Clinical Extreme	Extreme Age (Old)	65-year-old patient	Valid assessment within probability bounds	Pass
**Mathematical Validation (n=2)**					
10	Mathematical	Logit Probability Bounds	Test across best/moderate/worst case profiles	All probabilities $0 < p < 1$ for both linear and logit methods	Pass
11	Mathematical	Method Consistency	Compare linear vs. logit calculation methods	Both methods produce identical relative rankings	Pass
**Mechanism Diversity (n=2)**					
12	Mechanism	Redundancy Prevention	Cisgender woman with transport, childcare, mistrust barriers	Top 3 recommendations use $\geq$2 distinct mechanism categories	Pass
13	Mechanism	Tag Coverage	MSM on oral PrEP, best case scenario	All recommendations have mechanism tags assigned	Pass
**Data Export (n=2)**					
14	Export	JSON Structure Validity	Standard MSM patient profile	JSON output contains all required keys; serializable without error	Pass
15	Export	Explanatory Fields	PWID with housing/transportation barriers	JSON includes attrition factors, delay factors, and rationale for each recommendation	Pass
**Error Handling (n=3)**					
16	Error	Invalid Population	Attempt to use “INVALID_POP” as population	Raises ConfigurationError	Pass
17	Error	Invalid Barrier	Attempt to use “INVALID_BARRIER”	Raises ConfigurationError	Pass
18	Error	Invalid Setting	Attempt to use “INVALID_SETTING” as healthcare setting	Raises ConfigurationError	Pass

https://doi.org/10.3390/v18020237

S9.2 Summary Statistics

Table S10. Edge Case Testing Summary by Category

Category	Tests	Passed	Pass Rate
Clinical Edge Cases	9	9	100%
Mathematical Validation	2	2	100%
Mechanism Diversity	2	2	100%
Data Export	2	2	100%
Error Handling	3	3	100%
**Total**	**18**	**18**	**100%**

S9.3 Clinical Significance of Edge Case Validation

The 100% pass rate across 18 edge cases validates the following aspects of algorithmic robustness:

1. **Boundary condition handling:** Algorithm correctly processes extreme barrier loads (7+ barriers) and zero-barrier scenarios without mathematical failures.
2. **Population coverage:** All 7 population categories (MSM, cisgender women, transgender women, adolescents, PWID, pregnant/lactating, general) produce valid assessments.
3. **Age spectrum:** Full age range (16–65 years) handled correctly.
4. **Probability validity:** All predictions remain within valid probability bounds $(0 < p < 1)$ regardless of input extremity.
5. **Method equivalence:** Linear and logit-space calculations produce consistent relative rankings.
6. **Intervention diversity:** Mechanism diversity scoring successfully prevents redundant recommendations.
7. **Data integrity:** JSON export maintains complete structure for reproducibility and integration.
8. **Graceful error handling:** Invalid inputs trigger appropriate configuration errors rather than silent failures.

S9.4 Test Execution Details

Tests were executed using the pytest framework:

```
pytest test_edge_cases.py -v --tb=short
```

Complete test suite available in GitHub repository: https://github.com/Nyx-Dynamics/lai-prep-bridge-tool-pub
Test file: `test_edge_cases.py` (525 lines, 18 test functions across 5 test classes)

Note: Edge case testing validates computational robustness—that the algorithm handles extreme inputs without failure. It does not validate clinical accuracy of predictions for these extreme scenarios, which requires prospective validation with real patients.

Reference: A.C Demidont, DO(2025). Computational Validation of a Clinical Decision Support Algorithm for Long-Acting Injectable PrEP Bridge Period Navigation at UNAIDS Global Target Scale. *Viruses*

==

References, variant A: external bibliography

1. Landovitz, R.J.; Donnell, D.; Clement, M.E.; Hanscom, B.; Cottle, L.; Coelho, L.; et al. Cabotegravir for HIV prevention in cisgender men and transgender women. *New England Journal of Medicine* **2021**, *385*, 595–608. https://doi.org/10.1056/NEJMoa2101016.
2. Delany-Moretlwe, S.; Hughes, J.P.; Bock, P.; Ouma, S.G.; Hunidzarira, P.; et al. Cabotegravir for the prevention of HIV-1 in women: Results from HPTN 084. *The Lancet* **2022**, *399*, 1779–1789. https://doi.org/10.1016/S0140-6736(22)00538-4.
3. Bekker, L.G.; Das, M.; Abdool Karim, Q.; et al. Twice-yearly lenacapavir or daily F/TAF for HIV prevention in cisgender women. *New England Journal of Medicine* **2024**, *391*, 1179–1192. https://doi.org/10.1056/NEJMoa2407001.
4. Kelley, C.F.; Acevedo-Quiñones, M.; Agwu, A.L.; et al. Twice-yearly lenacapavir for HIV prevention in men and gender-diverse persons. *New England Journal of Medicine* **2025**, *392*, 1261–1276. https://doi.org/10.1056/NEJMoa2411858.
5. Altamirano, J.A.; Shukla, P.; Barnett, S.K. 1531. Early real-world experience of long-acting cabotegravir (CAB) for HIV pre-exposure prophylaxis (PrEP) in a large community-based clinic network (CAN Community Health): Utilization and PrEP persistence. *Open Forum Infectious Diseases* **2023**, *10*, ofad500.1366. https://doi.org/10.1093/ofid/ofad500.1366.
6. Hosek, S.G.; Landovitz, R.J.; Kapogiannis, B.; et al. Safety and feasibility of antiretroviral preexposure prophylaxis for adolescent men who have sex with men. *JAMA Pediatrics* **2017**, *171*, 1063. https://doi.org/10.1001/jamapediatrics.2017.2007.
7. Mistler, C.B.; Copenhaver, M.M.; Shrestha, R. The pre-exposure prophylaxis (PrEP) care cascade in people who inject drugs: A systematic review. *AIDS and Behavior* **2021**, *25*, 1490–1506. https://doi.org/10.1007/s10461-020-02988-x.
8. Biello, K.B.; Edeza, A.; Salhaney, P.; Biancarelli, D.L.; Mimiaga, M.J.; Drainoni, M.L.; Childs, E.; Bazzi, A.R. A missing perspective: injectable pre-exposure prophylaxis for people who inject drugs. *AIDS Care* **2019**, *31*, 1214–1220. https://doi.org/10.1080/09540121.2019.1587356.
9. UNAIDS. Global AIDS Update 2024: The Path That Ends AIDS, 2024.
10. Chin, M.H.; Clarke, A.R.; Nocon, R.S.; et al. A roadmap and best practices for organizations to reduce racial and ethnic disparities in health care. *Journal of General Internal Medicine* **2012**, *27*, 992–1000. https://doi.org/10.1007/s11606-012-2082-9.
11. Starbird, L.E.; DiMaina, C.; Sun, C.A.; Han, H.R. A systematic review of interventions to minimize transportation barriers among people with chronic diseases. *Journal of Community Health* **2019**, *44*, 400–411. https://doi.org/10.1007/s10900-018-0572-3.
12. Seyedroudbari, S.; Ghadimi, F.; Grady, G.; et al. Structural racism and discrimination along the PrEP continuum: A systematic review. *AIDS and Behavior* **2024**, *28*, 3001–3037. https://doi.org/10.1007/s10461-024-04387-y.
13. Lancaster, K.E.; Endres-Dighe, S.; Sucaldito, A.D.; Piscalko, H.; Madhu, A.; Kiriazova, T.; Batchelder, A.W. Measuring and Addressing Stigma Within HIV Interventions for People Who Use Drugs: a Scoping Review of Recent Research. *Current HIV/AIDS Reports* **2022**, *19*, 301–311. https://doi.org/10.1007/s11904-022-00619-9.
14. Sullivan, P.S.; Mena, L.; Elopre, L.; Siegler, A.J. Implementation Strategies to Increase PrEP Uptake in the South. *Current HIV/AIDS Reports* **2019**, *16*, 259–269. https://doi.org/10.1007/s11904-019-00447-4.
15. Patel, P.; Celum, C.; Bekker, L.G. Implementation of HIV prevention strategies globally. *The Lancet HIV* **2023**, *10*, e492–e494. Comment, https://doi.org/10.1016/S2352-3018(23)00139-X.
16. Shade, S.B.; Kirby, V.B.; Stephens, S.; Moran, L.; Charlebois, E.D.; Xavier, J.; Cajina, A.; Steward, W.T.; Myers, J.J. Outcomes and costs of publicly funded patient navigation interventions to enhance HIV care continuum outcomes in the United States: A before-and-after study. *PLOS Medicine* **2021**, *18*, e1003418. https://doi.org/10.1371/journal.pmed.1003418.
17. Spinelli, M.A.; Scott, H.M.; Vittinghoff, E.; et al. A panel management and patient navigation intervention is associated with earlier PrEP initiation in a safety-net primary care health system. *Journal of Acquired Immune Deficiency Syndromes* **2018**, *79*, 347–351. https://doi.org/10.1097/QAI.0000000000001801.

18. Kamitani, E.; Higa, D.H.; Crepaz, N.; et al. Identifying best practices for increasing linkage to, retention in, and reengagement with HIV medical care: A systematic review. *AIDS and Behavior* **2024**, *28*, 2340–2349. https://doi.org/10.1007/s10461-024-04332-z.
19. Paskett, E.D.; Harrop, J.P.; Wells, K.J. Patient navigation: An update on the state of the science. *CA: A Cancer Journal for Clinicians* **2011**, *61*, 237–249. https://doi.org/10.3322/caac.20111.
20. Crooks, N.; Donenberg, G.; Matthews, A. Barriers to PrEP uptake among Black female adolescents and emerging adults. *Preventive Medicine Reports* **2023**, *31*, 102092. https://doi.org/10.1016/j.pmedr.2022.102092.
21. Walters, S.M.; Kral, A.H.; Simpson, K.A.; Wenger, L.; Bluthenthal, R.N. HIV pre-exposure prophylaxis prevention awareness, willingness, and perceived barriers among people who inject drugs in Los Angeles and San Francisco, CA, 2016–2018. *Substance Use and Misuse* **2020**, *55*, 2409–2419. https://doi.org/10.1080/10826084.2020.1823419.
22. Strathdee, S.A.; Kuo, I.; El-Bassel, N.; et al. Preventing HIV outbreaks among people who inject drugs in the United States: plus ça change, plus c'est la même chose. *AIDS* **2020**, *34*, 1997–2005. https://doi.org/10.1097/QAD.0000000000002673.
23. Randolph, S.D.; Johnson, R. A Salon-Based Intervention to Improve PrEP Uptake among Black Women. *New England Journal of Medicine* **2024**, *390*, 776–777. https://doi.org/10.1056/NEJMp2313708.
24. Deutsch, M.B.; Glidden, D.V.; Sevelius, J.; et al. HIV pre-exposure prophylaxis in transgender women: A subgroup analysis of the iPrEx trial. *The Lancet HIV* **2015**, *2*, e512–e519. https://doi.org/10.1016/S2352-3018(15)00206-4.
25. Natale-Pereira, A.; Enard, K.R.; Nevarez, L.; Jones, L.A. The role of patient navigators in eliminating health disparities. *Cancer* **2011**, *117*, 3543–3552. https://doi.org/10.1002/cncr.26264.
26. Haser, G.C.; Balter, L.; Gurley, S.; Thomas, M.; Murphy, T.; et al. Early implementation of long-acting injectable cabotegravir/rilpivirine at Ryan White clinics in the U.S. South. *AIDS Research and Human Retroviruses* **2024**, *40*, 690–700. https://doi.org/10.1089/AID.2024.0007.
27. World Health Organization. *Task Shifting: Rational Redistribution of Tasks among Health Workforce Teams*; World Health Organization: Geneva, Switzerland, 2008.
28. Violette, L.R.; Zewdie, K.; Gitahi, N.; Beima-Sofie, K.; Heffron, R. The pathway to delivering injectable cabotegravir for HIV prevention: A review of implementation considerations. *Implementation Science Communications* **2024**, *5*, 101. https://doi.org/10.1186/s43058-024-00637-1.
29. Rousseau, E.; Julies, R.F.; Madubela, N.; Kassim, S. Novel platforms for biomedical HIV prevention delivery to key populations. *Current HIV/AIDS Reports* **2021**, *18*, 500–507. https://doi.org/10.1007/s11904-021-00578-7.
30. Touger, R.; Wood, B.R. A review of telehealth innovations for HIV pre-exposure prophylaxis (PrEP). *Current HIV/AIDS Reports* **2019**, *16*, 113–119. https://doi.org/10.1007/s11904-019-00430-z.
